# Yishen Tongbi decoction attenuates inflammation and bone destruction in rheumatoid arthritis by regulating JAK/STAT3/SOCS3 pathway

**DOI:** 10.3389/fimmu.2024.1381802

**Published:** 2024-06-20

**Authors:** Jia Xu, Wei Jiao, Dan-Bin Wu, Jia-Hui Yu, Li-Juan Liu, Ming-Ying Zhang, Guang-Xing Chen

**Affiliations:** ^1^ First Clinical Medical School, Guangzhou University of Chinese Medicine, Guangzhou, China; ^2^ Department of Rheumatology, The First Affiliated Hospital of Guangzhou University of Chinese Medicine, Guangzhou, China; ^3^ Baiyun Hospital of The First Affiliated Hospital of Guangzhou University of Chinese Medicine, Guangzhou, China

**Keywords:** Yishen-Tongbi decoction, rheumatoid arthritis, inflammation, collagen-induced arthritis, RAW264.7 macrophages

## Abstract

**Background:**

Yishen-Tongbi Decoction (YSTB), a traditional Chinese prescription, has been used to improve syndromes of rheumatoid arthritis (RA) for many years. Previous research has shown that YSTB has anti-inflammatory and analgesic properties. However, the underlying molecular mechanism of the anti-RA effects of YSTB remains unclear.

**Purpose and study design:**

The purpose of this research was to investigate how YSTB affected mice with collagen-induced arthritis (CIA) and RAW264.7 cells induced with lipopolysaccharide (LPS).

**Results:**

The findings show that YSTB could significantly improve the clinical arthritic symptoms of CIA mice (mitigate paw swelling, arthritis score, thymus and spleen indices, augment body weight), downregulated expression of pro-inflammatory cytokines like tumor necrosis factor-alpha (TNF-α), interleukin-1β (IL-1β), IL-6 and IL-17, while upregulated the level of anti-inflammatory like IL-10 and transforming growth factor-β (TGF-β). Meanwhile, YSTB inhibits bone erosion and reduces inflammatory cell infiltration, synovial proliferation, and joint destruction in CIA mice. In addition, we found that YSTB was able to suppress the LPS-induced inflammation of RAW264.7 cells, which was ascribed to the suppression of nitric oxide (NO) production and reactive oxygen species formation (ROS). YSTB also inhibited the production of inducible nitric oxide synthase and reduced the releases of pro-inflammatory cytokines TNF-α, IL-1β, and IL-6 in LPS-induced RAW264.7 cells. Furthermore, the phosphorylation expression of JAK2, JAK3, STAT3, p38, ERK and p65 protein could be suppressed by YSTB, while the expression of SOCS3 could be activated.

**Conclusion:**

Taken together, YSTB possesses anti-inflammatory and prevention bone destruction effects in RA disease by regulating the JAK/STAT3/SOCS3 signaling pathway.

## 1 Introduction

Rheumatoid arthritis (RA), an “immortal cancer”, is an autoimmune chronic disease characterized by progressive symmetrical joint destruction, deformity, incapacity, and even premature death (1). Approximately 1.0% of the world’s population suffers from RA, and the prevalence is higher in women aged 40–60 years (2, 3). There is currently no medicine that can cure RA. Non-steroidal anti-inflammatory drugs (NSAIDs), disease-modifying anti-rheumatic drugs (DMARDs), Glucocorticoids (GC), and targeted biologics are used to ameliorate the condition (4). Nonetheless, these drugs have some side effects, including herpes zoster infection, venous thromboembolism (VTE) risk, neutropenia and lymphopenia, headache, nausea, and gastrointestinal discomfort, and the patient’s illness remission is still far below the desired level (5). In recent years, molecularly targeted therapies have become a hot spot for research, and JAK is undoubtedly one of the most popular targets. Although several biologics targeting JAK kinase have been approved for the treatment of RA, their long-term efficacy and safety need to be further evaluated, as some patients still show poor response and resistance to biologics, and the potential risk of infection is high and expensive (6). Thus, there is an urgent demand to develop low-side effects and cost-effective drugs for the long-term treatment of RA.

Although the exact pathogenesis of RA is intricate and vague, it is generally acknowledged that inflammation is a crucial pathological characteristic of RA (7). For instance, tumor necrosis factor-alpha (TNF-α) induces activation of leukocytes and endothelial cells and amplifies cytokine and chemokine responses (8). Interleukin-1β (IL-1β) is involved in synovial inflammation and synovial fibrosis in RA (9). IL-6 induces the differentiation and formation of osteoclasts and promotes angiogenesis and the degradation of bone and cartilage (10). IL-17 induces joint inflammation and promotes angiogenesis (11). What’s more, IL-6 can promote the synergistic function of TNF-α and IL-1β to further aggravate joint inflammation and promote the establishment and maintenance of a synovial inflammatory environment (12). Previous research confirms that these pro-inflammatory factors are abundant in the serum and synovial fluid of patients with RA and are associated with the degree of RA disease activity (13). In contrast, IL-10 and TGF-β are potent anti-inflammatory cytokines. IL-10 regulates the production of endogenous proinflammatory cytokines in the synovial tissue of RA (14). Of note, these inflammatory cytokines are strictly regulated through the Janus kinase/signal transducer and activator of transcription (JAK/STAT) signaling pathway in RA patients (15). Contrarily, the suppressor of cytokine signaling 3 (SOCS3) plays a negative regulatory factor role in the JAK/STAT pathway, which can restrain the generation of pro-inflammatory mediators by inhibiting the phosphorylation of STAT3 (16). In addition, NF-κB and MAPK pathways have been shown to play important roles in cytokine-induced inflammation and tissue destruction. Therefore, targeting modulate the JAK-STAT as well as the MAPK/NF-κB key phosphorylated proteins to decrease the expression of inflammatory cytokines might be a promising strategy in RA treatment.

Traditional Chinese medicine (TCM) is China’s traditional unique advantage. Increasing evidence shows that TCM can become a new therapeutic strategy for RA with analgesic and anti-inflammatory effects. Yishen-Tongbi decoction (YSTB) is composed of Tripterygium hypoglaucum (Levl.) Hutch(gymnosporia; Kunmingshanhaitang), Herba ecliptae (composite famility; Mohanlian), Fructus lycii (Solanaceae; Gouqizi), Fructus Ligustri Lucidi (olive family; Nvzhenzi), Eucommia ulmoides Oliver (eucommiaceae; Duzhong) and Salvia miltiorrhiza Bge (labiatae; Danshen). A previous clinical trial showed that YSTB has a good effect on RA patients according to relieving joint swelling and pain, shortening morning stiffness time, and reducing inflammatory levels such as erythrocyte sedimentation rate (17). Meanwhile, YSTB can regulate immune balance by inhibiting excessive B cell activation in ST486 cells (18). Furthermore, YSTB has been reported to significantly decrease and relieve acute inflammation in the adjuvant arthritis rat model, protecting joints from destruction. Based on the above research background, the group hypothesized that YSTB might achieve improvement in RA based on its good anti-inflammatory and immunomodulatory effects. Nevertheless, the efficacy of YSTB in RA still calls for verification with increasing research, and the anti-inflammatory and anti-bone destruction mechanisms require further study unveiled. Thus, this study investigated the efficacy and mechanism of action of YSTB in RA by constructing collagen-induced arthritis (CIA) mice model and RAW264.7 cell model, so as to provide a scientific theoretical basis for the clinical application of YSTB in the treatment of RA.

## 2 Materials and methods

### 2.1 Drug, reagents and preparation of YSTB

Details of drugs and reagents are shown in **Supplementary File** section *a*). The First Affiliated Hospital of Guangzhou University of Chinese Medicine provided the Yishen-tongbi decoction (YSTB) (Guangdong, China).

YSTB was composed of Tripterygium hypoglaucum (Levl.) Hutch (25g), Eucommia ulmoides Oliver (15g), Fructus lycii (15g), Salvia miltiorrhiza Bge (15g), Herba ecliptae (15g) and Fructus Ligustri Lucidi (15g). Tripterygium hypoglaucum (Levl.) Hutch was soaked 10 times in pure water for 30 min and boiled for half an hour. The other medicine was added and cooked for 3.5h, and then the other herbs were added and cooked for another 30min. Using a rotary evaporator at 60°C, the filtrate was concentrated and filtered, and then frozen-dried to create a lyophilized powder for use *in vitro* and *in vivo* studies.

### 2.2 Animals

We purchased forty male DBA/1 mice (8 weeks old, 20 ± 2 g) from Beijing Vital River Laboratory Animal Technology Co., Ltd. (Beijing, China). The Animal Laboratory Research Ethics Committee of the First Affiliated Hospital of Guangzhou University of Chinese Medicine approved this experimental procedure. Mice were placed in a 12h light/dark cycle regulatory environment for 7 days before the experiment to adapt. The room temperature for feeding was controlled at 23 ± 2°C, relative humidity 50–70%, and ventilation was appropriate.

### 2.3 Induction of collagen-induced arthritis model and drug treatment

**Figure 1:**
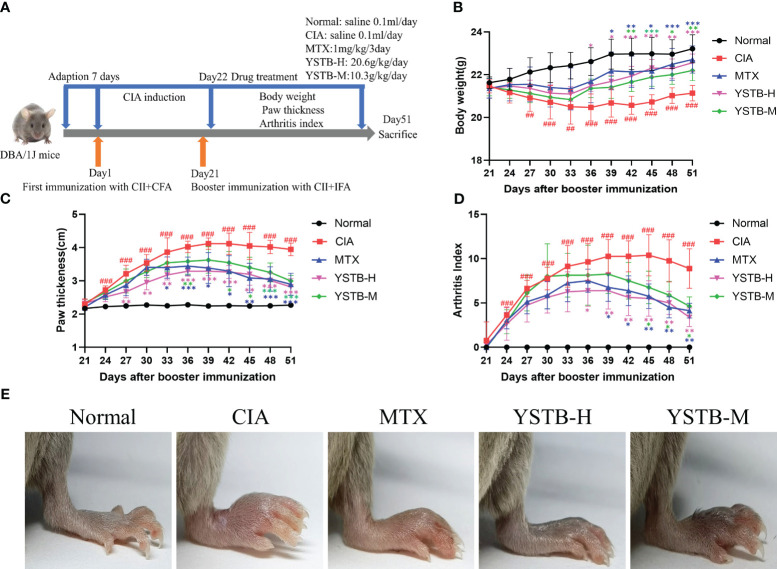
Effects of YSTB on the severity of arthritis in CIA mice. **(A)** Schematic diagram of the experimental process of the CIA mice. The body weight **(B)** and paw thickness **(C)** and arthritis index **(D)** of each group of mice were measured and scored every 3 days after booster immunization. **(E)** Representative image of each group of mice hind limbs taken on day 51. Data are expressed as the mean ± SD (n=8). Compared with the normal group, ##*P* < 0.01, ###*P* < 0.001; compared with the CIA model group, *P < 0.05, **P < 0.01 and ***P < 0.001.

The CIA model was established based on previous literature protocol (19) and described in detail in **Supplementary File** section *b*). The flow chart of induction and treatment of CIA mice is shown in **Figure 1A**.

### 2.4 Assays

Detailed information on Hematoxylin-Eosin and Safranin O Staining, Immunohistochemistry Analysis, Cell Culture and Cell Morphology Changes, Cell Viability Assay, Detection of ROS, NO and cytokine, Western-blot analysis, qRT-PCR, Arthritic Severity Scores of CIA, Determination of Serum Cytokine Concentrations, Thymus and Spleen Index Assay, Micro CT Scanning Analysis was shown in **Supplementary File** section *c-n*).

### 2.4 Statistical analysis

All data were expressed as mean ± standard deviation (SD) and analyzed by SPSS 26.0. Consistent with a normal distribution, one-way ANOVA was used to test. Then, Tukey’s test for homogeneity of variances, and Tamhane’s T2 test for unequal variances. Not conforming to a normal distribution, using the Kruskal-Wallis test. *P* < 0.05 was deemed statistically significant. GraphPad Prism 8 software was applied to draw the graphs.

## 3 Results

### 3.1 YSTB improves severity of arthritis in CIA mice

To assess the efficacy of YSTB in the treatment of RA, we decided to use CIA mice, which have pathological and immunological features similar to human RA (20). MTX can mitigate the symptoms of RA and reduce joint damage, and its efficacy in the clinical treatment of RA has been affirmed. Consequently, this experiment used MTX as a positive control drug. The onset characteristics of arthritis after modeling appear within one to two weeks after the booster immunization. As illustrated in **Figure 1E**, CIA mice have been successfully modeled, and obvious joint swelling and erythema can be clearly noticed, whereas the severity degree of arthritis in mice treated with YSTB or MTX is significantly attenuated. The paw thickness and arthritis index of the CIA model group increased on the 24th day (**Figures 1C, D**), the 27th day weight loss initially appeared (**Figure 1B**), which indicates that the CIA mice were successfully modeled (*P* < 0.001, *P* < 0.001, *P* < 0.01, respectively). In comparison to the CIA model group, the mice’s arthritis index as well as the severity of their paw swelling, improved in a dose-dependent manner after receiving the treatment with MTX or YSTB. At the same time, MTX and YSTB groups regain weight from the 36th day, especially in the YSTB-H group. These findings suggest that YSTB has potent anti-rheumatoid properties.

### 3.2 YSTB modulates the levels of inflammatory cytokines in serum of CIA mice

**Figure 2:**
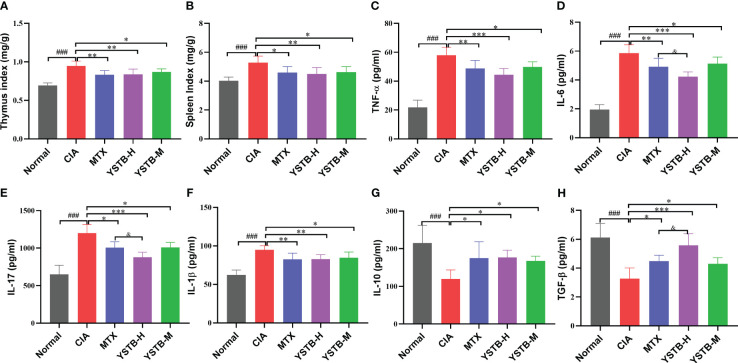
Effects of YSTB on the organ index and serum levels of inflammatory cytokines in CIA mice. **(A)** Thymus index. **(B)** Spleen index. The level of pro-inflammation cytokines TNF-α **(C)**, IL-6 **(D)**, IL-17 **(E)**, IL-1β **(F)**, anti-inflammation cytokines lL-10 **(G)** and TGFβ **(H)** in the serum of CIA mice were detected by ELISA. Data are expressed as the mean ± SD (n=8). Compared with the normal group, ###*P* < 0.001; compared with the CIA model group, *P < 0.05, **P < 0.01 and ***P < 0.001; compared with the MTX group, ^&^ *P* < 0.05.

Cytokines are important molecules participant in the immune-inflammatory response to RA. Consequently, we used ELISA kits to detect the levels of different inflammatory cytokines in mice serum. The data showed that TNF-α, IL-6, IL-17 as well as IL-1β, both indicators of pro-inflammatory, were relatively elevated in the serum of mice in the CIA model group (*P* < 0.001) (**Figures 2C–F**). On the contrary, indicators of anti-inflammatory IL-10 and TGF-β were relatively decreased (*P* < 0.001) (**Figures 2G, H**). TNF-α, IL-6, IL-17, and IL-1β levels were down-regulated after YSTB or MTX intervention, while IL-10 and TGF-β levels were up-regulated. Surprisingly, the YSTB-H group’s effect was comparable to that of the MTX group and even better in terms of lowering IL-6 and IL-17 while boosting TGF-β levels than MTX. The results showed that YSTB could alleviate the inflammatory response *in vivo* by increasing the synthesis of anti-inflammatory substances while decreasing the production of pro-inflammatory factors.

### 3.3 YSTB reduced the thymus and spleen index of CIA mice

The thymus and spleen are both known to be essential immune organs, and their organ indices can, to some extent, reflect the body’s immune function. As illustrated in (**Figures 2A, B**), as opposed to the normal group, the thymus and spleen indices of CIA model mice were manifestly raised after modeling, while diminished in the MTX group and YSTB group, which could demonstrate that the CIA model group mice developed hyperimmune responses after induction, whereas YSTB and MTX could inhibit abnormal immune function.

### 3.4 YSTB attenuated the histopathological changes in CIA mice

**Figure 3:**
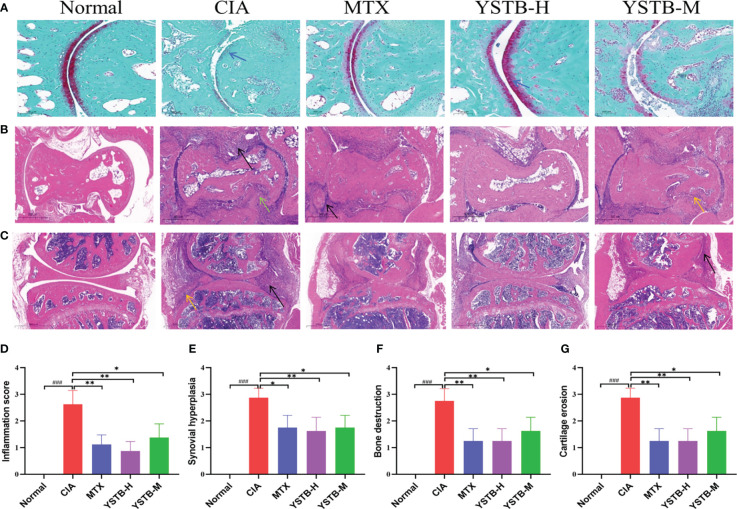
Effects of YSTB on the histologic changes in CIA mice (200×). Safranin O staining of the ankle joint surfaces of mice **(A)** (scale bar 100μm). Hematoxylin and eosin (HE) were used for the ankle joint **(B)** and knee joint **(C)** of mice of representative sections that were stained (scale bar 500μm). Histological score: inflammation score **(D)**, synovial proliferation **(E)**, bone destruction **(F)**, cartilage erosion **(G)**. Data are expressed as the mean ± SD (n=8). Infiltrated inflammatory cells are denoted by the black arrow, bone erosion by the yellow arrow, cartilage rupture indicated by a blue arrow and synovial hyperplasia in distinct parts by the green arrow. Compared with the normal group, ###*P* < 0.001; compared with the CIA model group, *P < 0.05, **P < 0.01 and compared with the MTX group.

The Safranin-O staining results show that the normal undamaged articular surface is clearly green and the cartilage is bright red between the joints. In the CIA model group, the cartilage layer became thinner and the cartilage red was not colored. The cartilage layer thickness increased in MTX and YSTB groups, and the red color was augmented (**Figure 3A**). After successful modeling of CIA mice, apparent synovial hyperplasia appeared in ankle joint (**Figure 3B**) and knee joint (**Figure 3C**), fibroblast-like synovial cells (FLS) were disorderly and loosely arranged, a massive number of inflammatory cells were infiltrated, bone and cartilage were severely eroded. In comparison to the CIA model group, YSTB or MTX treatment meaningfully decreased the levels of inflammatory cell infiltration, bone destruction, synovial proliferation, and cartilage erosion (**Figures 3D–G**). It is worth noting that the YSTB-H group was more effective than the MTX group in reducing inflammatory cell infiltration and bone destruction. These results suggest that YSTB strongly inhibited inflammation, bone destruction, and cartilage damage in the joint tissue of CIA mice.

### 3.5 YSTB ameliorates bone destruction and loss in CIA mice

**Figure 4:**
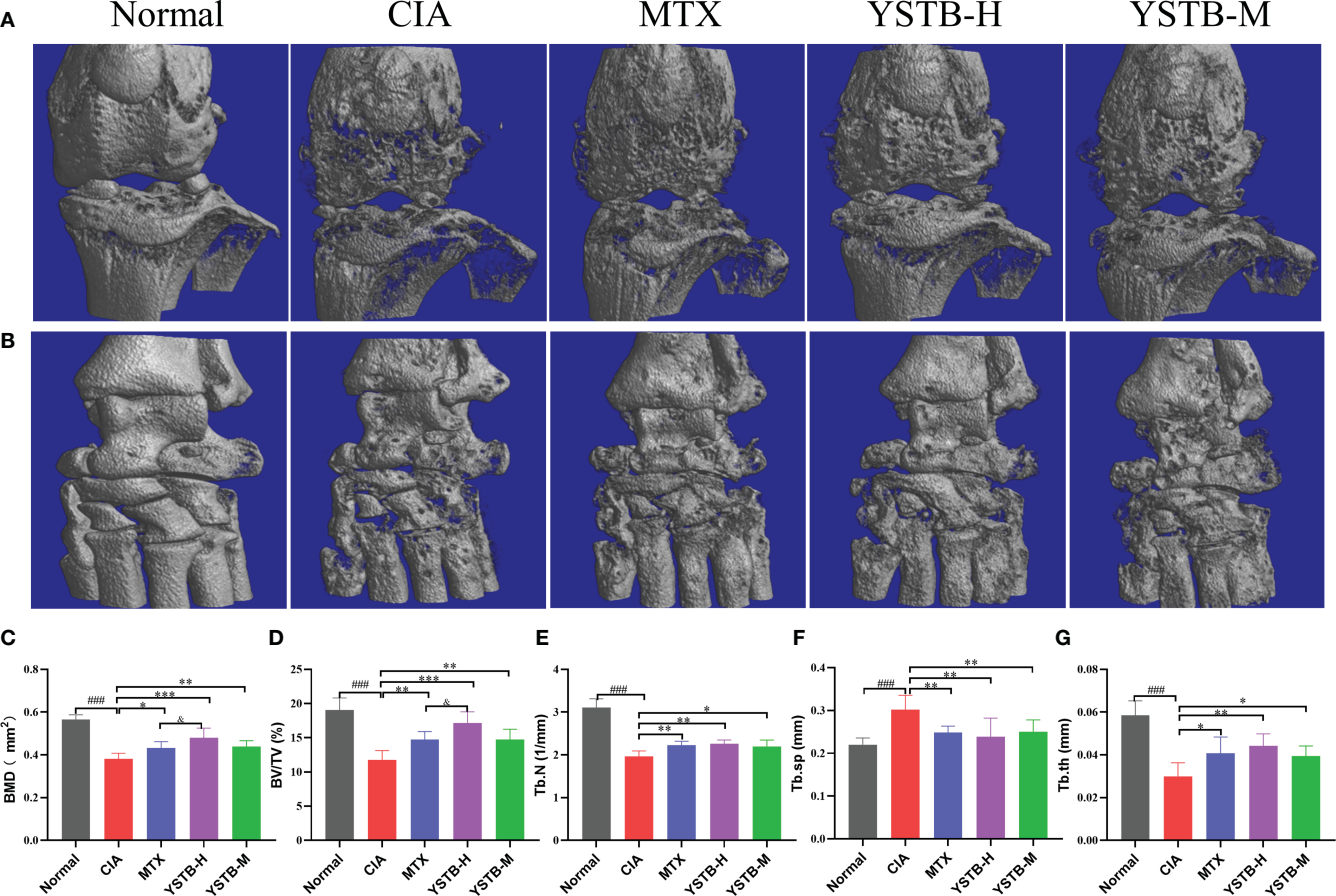
Effects of YSTB on articular bone erosion in CIA mice. Representative micro-CT images of the knee joint **(A)** and ankle joint **(B)** surface of each group of mice. evaluated of joint destruction: Bone mineral density (BMD) **(C)**. Bone volume fraction (BV/TV) **(D)**. Trabecular bone number (Tb.N) **(E)**. Trabecular bone space (Tb.Sp) **(F)**. Trabecular bone thickness (Tb.Th) **(G)**. Data are expressed as the mean ± SD (n=8). Compared with the normal group, ###*P* < 0.001; compared with the CIA model group, *P < 0.05, **P < 0.01 and ***P < 0.001; compared with the MTX group, ^&^ *P* < 0.05.

To verify the function of YSTB on the destruction of joints and bones of CIA mice, the knee and ankle joints of all groups were scanned by micro-CT. As exemplified in (**Figures 4A, B**), the joint surface of normal mice is clear and smooth without abnormal changes, while the CIA model mice exhibited rough cartilage surface, severe bone erosion and narrowed joint space. It can be seen from the quantitative indicators that the Tb.N, BMD, BV/TV and Tb.Th values are pronounced heighten meanwhile the Tb.Sp value is considerably declined (**Figures 4C–G**). These indicators have improved to varying degrees after MTX or YSTB group intervention. Remarkably, the improvement is more obvious in BMD and BV/TV with the YSTB-H group treatment. These data reveal that YSTB treatment can alleviate the degree of bone damage in CIA mice to a certain extent, thereby preventing further destruction of joint tissues.

### 3.6 YSTB regulation the expression of p-JAK2, p-JAK3, p-STAT3 and SOCS3 in the ankle joints of CIA mice

**Figure 5:**
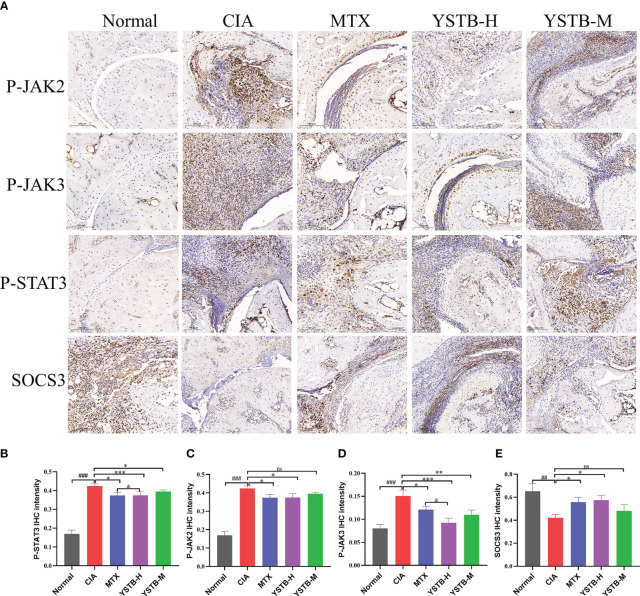
Effects of YSTB on the expression of p-JAK2, p-JAK3, p-STAT3, SOCS3 in CIA mice. **(A)** Representative immunohistochemical staining images of p-JAK2, p-JAK3, p-STAT3, SOCS3 in ankle joints of mice in different groups (scale bar 100μm). The quantification of p-JAK2 **(B)**, p-JAK3 **(C)**, p-STAT3 **(D)** and SOCS3 **(E)** expression in the ankle joints of mice in each group were evaluated by Image pro plus 6.0, respectively. Data are expressed as the mean ± SD (n=3). Compared with the normal group, ##*P* < 0.01, ###*P* < 0.001; compared with the CIA model group, *P < 0.05, **P < 0.01, ***P < 0.001; compared with the MTX group, *^&^P < 0.05*. ns, no significance.

As demonstrated in **Figure 5A**, IHC detected the expressions of p-JAK2, p-JAK3, p-STAT3, and SOCS3 with different groups of CIA mice. When compared to the normal group, the expression distribution of p-JAK2, p-JAK3 and p-STAT3 positive cells (brown or brownish-yellow) were more extensive and abundant, while the expression distribution of SOCS3 positive cells was diminished. In the MTX or YSTB-H intervention group, the positive expression levels of p-JAK2, p-JAK3, and p-STAT3 showed different degrees of decreasing trend when compared to the CIA group (**Figures 5B–D**), yet the expression of SOCS3 was enhanced (**Figure 5E**).

### 3.7 Morphological changes of RAW264.7 cells and YSTB effects on cell viability

**Figure 6:**
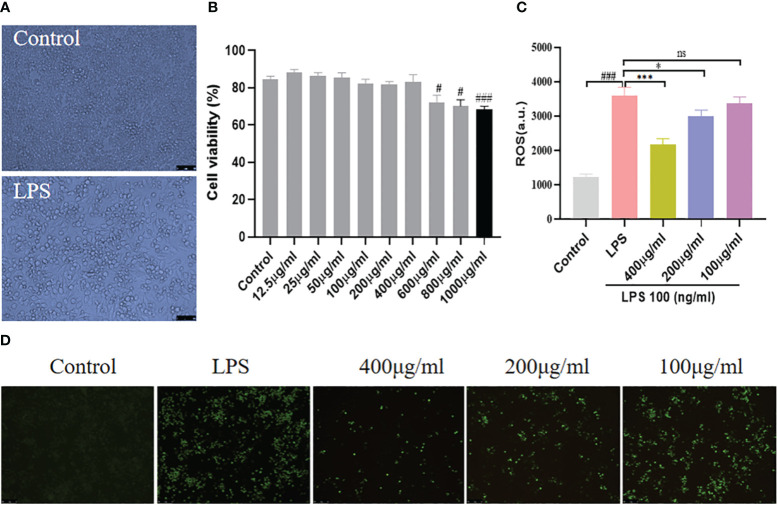
Effect of YSTB on cell viability and expression of ROS in RAW264.7 cells. **(A)** The morphological changes of RAW264.7 cells, the cells differentiated and pseudopodia appeared with LPS stimulation. **(B)** CCK8 assays the cell viability. The RAW264.7 cells were pretreated with YSTB (100, 200, 400μg/mL) for 2h. Afterward, cells were stimulated with LPS (100ng/mL) for 24h. **(C)** Fluorescence intensity of ROS. **(D)** Representative images of ROS expression photographs were taken by fluorescence microscopy. Data are expressed as the mean ± SD (n=3). Compared with the control group, #*P* < 0.05, ###*P* < 0.001; compared with the LPS group, *P < 0.05, ***P < 0.001. ns, no significance.

To validate our findings from CIA mice, we looked into the anti-inflammatory properties of YSTB in the LPS-induced RAW264.7 model. LPS is a strong activator of cytokine production by immune cells. It can be seen from **Figure 6A** that the outline of normal RAW264.7 cells is clear, round, and plump. After LPS (100ng/ml) stimulation, the cells began to deform, showing long fusiform and antennae-like pseudopodia, and spread to the surroundings. Morphological changes present that LPS stimulation produces typical macrophage-related features of inflammation. In addition, RAW264.7 cells were determined for cell viability using the CCK8 method following various drug concentration interventions of YSTB to determine the optimal YSTB concentration for subsequent experiments. Compared with the Control group, YSTB showed a slight upward trend in cell viability at concentration doses of 12.5–50 μg/mL; there was no significant effect on the viability of RAW264.7 cells at concentrations less than 400 μg/mL concentration. However, RAW264.7 cell viability gradually decreased when the YSTB concentration range was 600–1000μg/mL, and the difference was statistically significant (*P*< 0.05, *P*< 0.05, *P*< 0.001, respectively). Accordingly, we set the concentrations of YSTB 100, 200, and 400μg/mL as the low, medium, and high dose groups for subsequent *in vitro* studies, respectively (**Figure 6B**).

### 3.8 YSTB inhibited intracellular ROS production in RAW264.7 cells

Our research found that RAW264.7 cells produced a large amount of endogenous ROS under the stimulation of LPS (100ng/ml), the intracellular green fluorescence was significantly enhanced, and the fluorescence intensity of ROS increased (**Figures 6C, D**). In intervention with YSTB, the intracellular green fluorescence gradually dimmed, and the ROS fluorescence intensity level was inhibited. Moreover, the 400μg/ml and 200μg/ml groups were more effective than the 100μg/ml group, especially the 400μg/ml group (*P*< 0.001, *P* < 0.05, respectively). These results manifest that YSTB can effectively eliminate the accumulation of endogenous ROS induced by LPS in RAW264.7 cells.

### 3.9 YSTB decreased LPS-stimulated release of NO and cytokines in RAW264.7 cells

**Figure 7:**
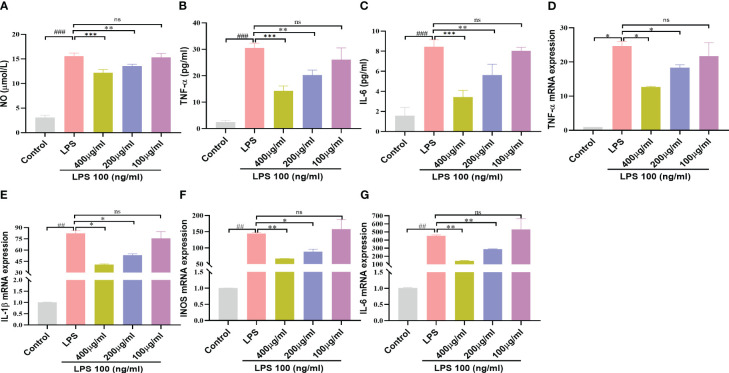
Effect of YSTB on Inflammatory mediators and Cytokines in LPS-induced RAW264.7 cells. **(A)** NO release measured by detecting nitrite content. The release of TNF-α **(B)**, IL-6 **(C)** and IL-1β **(D)** in cell culture supernatant was detected by ELISA, respectively. Relative mRNA expression levels of TNF-α **(E)**, IL-1β **(F)**, iNOS **(G)** and IL-6 **(H)** were determined by real-time PCR. Data are expressed as the mean ± SD (n=3). Compared with the control group, ##*P* < 0.01 and ###*P* < 0.001; compared with the LPS group, *P < 0.05, **P < 0.01 and ***P < 0.001. ns, no significance.

NO is closely related to the inflammatory and immune responses of macrophages (21). As shown in **Figure 7A**, the LPS (100ng/mL) induced group produced more NO than the control group, whereas doses of YSTB 400μg/ml and 200μg/ml dramatically suppressed the release of NO in a concentration-dependent manner, while 100μg/ml had no inhibitory effect (*P*< 0.001, *P* < 0.01, respectively). Meanwhile, TNF-α, IL-1β and IL-6 expression levels were increased by LPS stimulation. Interestingly, the 400 μg/ml of YSTB significantly decreased the expression of these inflammatory indicators in RAW264.7 cells, while the 100μg/ml dose of YSTB did not show any inhibitory effect (**Figures 7B–D**). These findings reveal that YSTB could ameliorate the inflammatory reaction of RAW264.7 cells stimulated with LPS within the experimental concentration range.

### 3.10 YSTB suppressed the LPS-stimulated mRNA expression of pro-inflammatory factors

We further implement RT-PCR experiments to determine the influence of YSTB on pro-inflammatory gene levels using LPS-induced RAW264.7 cells. As illustrated in **Figures 7D–G**, it could be seen that the YSTB group (200, 400μg/ml) had lower levels of IL-6, IL-1β, TNF-α and iNOS mRNA than the LPS group, whereas dose of 100μg/ml YSTB had no significant difference. These findings indicate that YSTB has potent inflammatory inhibitory properties in LPS-induced RAW264.7 cells, which is consistent with the results revealed by ELISA.

### 3.11 YSTB regulates the JAK/STAT3/SOCS3 and related signaling pathway in RAW264.7 cells

**Figure 8:**
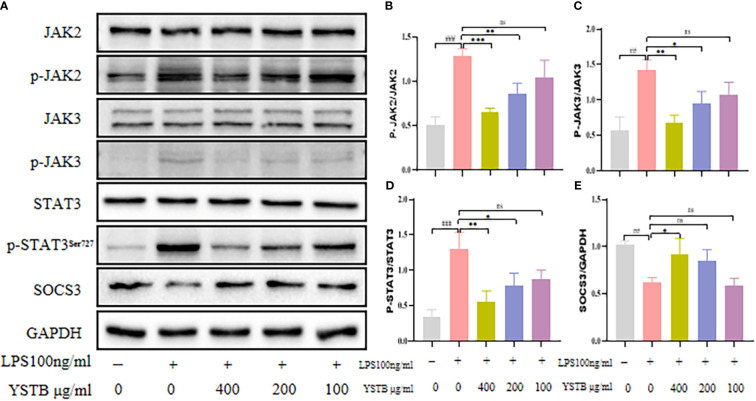
Effect of YSTB on the expression of JAK/STAT3/SOCS3 in LPS-induced RAW264.7 cells. **(A)**. The quantification of p-JAK2/JAK2 **(B)**, p-JAK3/JAK3 **(C)**, p-STAT3/STAT3 **(D)** and SOCS3/GAPDH **(E)** protein expression in the RAW264.7 cells in each group were evaluated by ImageJ, respectively. Data are expressed as the mean ± SD (n=3). Compared with the control group, ##*P* < 0.01, ###*P* < 0.001; compared with the LPS group, *P < 0.05, **P < 0.01 and ***P < 0.001. ns, no significance.

**Figure 9:**
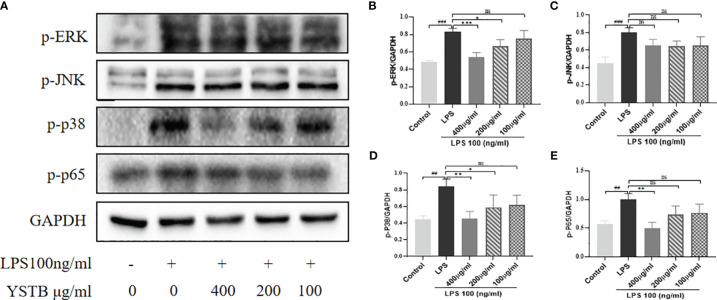
Effect of YSTB on the expression of p-JNK, p-p38, p-ERK and p-p65 in LPS-induced RAW264.7 cells. **(A)**. The quantification of p-ERK/GAPDH **(B)**, p-JNK/GAPDH **(C)**, p-p38/GAPDH **(D)** and p-p65/GAPDH **(E)** protein expression in the RAW264.7 cells in each group were evaluated by ImageJ, respectively. Data are expressed as the mean ± SD (n=3). Compared with the control group, ##*P* < 0.01, ###*P* < 0.001; compared with the LPS group, *P < 0.05, **P < 0.01 and ***P < 0.001. ns, no significance.

To elucidate the intrinsic mechanism of YSTB, we examined the expression of the JAK/STAT3/SOCS3 pathway (**Figure 8A**), as well as the expression of key phosphoproteins (p-JNK, p-p38, p-ERK, and p-p65) in RAW264.7 cells using Western blotting (**Figure 9A**, **Supplementary Material**). In comparison to the control group, the p-JAK2/JAK2, p-JAK3/JAK3, p-STAT3/STAT3 were all increased and SOCS3/GAPDH expression was decreased in the LPS model group. The decreased trend of p-JAK2/JAK2, p-JAK3/JAK3, p-STAT3/STAT3 were pronounced in the YSTB-H (400μg/ml) and YSTB-M (200μg/ml) group (**Figures 8B–D**). In contrast, SOCS3 expression was elevated merely in the YSTB (400μg/ml) group (**Figure 8E**). In the LPS model group, the expression of p-p65, p-p38 and p-ERK was significantly increased, and compared with the model group, the levels of p-p65, p-p38 and p-ERK decreased in a concentration-dependent manner in the group administered with YSTB, but there was no significant decreasing trend of p-JNK(**Figures 9B–E**, **Supplementary Material**).

## 4 Discussion

A six-month double-blind, randomized, controlled, non-inferiority trial of YSTB and methotrexate in active rheumatoid arthritis showed that the efficacy of YSTB monotherapy was not poorer than that of MTX monotherapy, and was even better after a short period of treatment (22). Next, the group will conduct a prospective, observational, multi-center cohort study for more in-depth validation (23). Previously, we used UPLC-Q-TOF-MS/MS to qualitatively analyze YSTB and identified 40 chemical components, including phenolic acids, flavonoids, alkaloids, triterpenes, et (18). Interestingly, most of these compounds have been proven to have anti-rheumatoid effects. As an example, triptolide is capable of lowering the proliferation, invasion, and inflammatory response of fibroblast-like synovial cells in rheumatoid arthritis. Danshensu has been shown to have anti-inflammatory, antioxidant and osteoblast differentiation modulating effects (24). Tanshinone IIA reduces the expression of pro-inflammatory cytokines in AA mice while also inhibiting synovial cell proliferation, migration, and invasion (25). Therefore, these compound components may be the effective material basis for the anti-RA effect of YSTB, and we can reasonably speculate that the YSTB compound has powerful anti-inflammatory effects.

To evaluate the therapeutic effect of YSTB, we used male DBA/1 mice to create a CIA model. CIA model mice lose appetite due to persistent inflammatory stimulation, immune system disturbances, and stress response after modeling. Therefore, the severity of rheumatoid arthritis can be reflected by the degree of paw swelling, arthritis scores, and body weight changes in mice. In our experiments, it can be seen that YSTB can reduce joint swelling and inflammatory index in mice and restore the body weight of mice. In RA disease, there is marked swelling of the thymus and spleen organs and weight gain due to a disturbance of the immune system. The thymus and spleen indices were significantly decreased after YSTB treatment, suggesting that YSTB has an immuno-suppressive effect on CIA mice. Histopathological results showed that synovial hyperplasia and inflammatory cell infiltration were obviously reduced, cartilage destruction and bone erosion were controlled treatment with YSTB. Moreover, micro-CT is currently the “gold standard” for evaluating the microstructure of small animal bone specimens and large animal isolated bone tissue. The micro-CT results exhibited that YSTB could greatly improve joint destruction in CIA mice, and increase Tb.N, BMD, BV/TV, Tb.Th while decreasing Tb.Sp. Notably, we found that the YSTB-H dose group (the clinically equivalent dose) was as effective or even better than MTX in improving RA symptoms.

Although the arthropathy mechanism of RA is not completely understood, inflammatory cytokines are thought to play a significant role in the pathophysiology of RA. Anti-inflammatory cytokines can postpone or even prevent inflammation and promote tissue repair, whereas pro-inflammatory cytokines are known to promote autoimmune inflammation and structural tissue damage. Serum TNF-α, IL-17, IL-1β, and IL-6 levels were significantly decreased in the YSTB treatment group, while TGF-β and IL-10 levels were increased. These results imply that YSTB has a significant effect on regulating inflammatory homeostasis in rheumatoid arthritis mice.

Meanwhile, macrophages have been linked to the pathogenesis of RA. Activated macrophages infiltrate RA patients’ synovial fluid and secrete pro-inflammatory cytokines (TNF-α, IL-1β, and IL-6), which in turn promote synovial inflammation and lead to cartilage and bone destruction, which correlates with the severity of RA. Additionally, NO is directly involved in tissue and joint damage in the pathogenesis of RA (26), and iNOS is the main active enzyme involved in cellular inflammation. Once macrophages are stimulated by LPS, the expression of iNOS can be induced, which in turn produces a large amount of NO, leading to aggravation of inflammation. Excessive NO results in vascular extravasation and altered membrane permeability, as well as the production of pro-inflammatory cytokines such as TNF-α, IL-6, and IL-1β. These pro-inflammatory factors can in turn induce the synthesis of iNOS, release more NO, and finally form a vicious circle (27). ROS are key mediators of oxidative stress and inflammation. Excessive secretion of ROS by macrophages can mediate tissue damage and affect the progression of RA. Therefore, inhibition of macrophage-mediated inflammatory responses may be a potential strategy to alleviate disease progression in RA. Based on these factors, we performed *in vitro* anti-inflammatory experiments using LPS-induced RAW264.7 mouse macrophages.

YSTB significantly inhibited NO secretion in RAW264.7 cell inflammation model by LPS-stimulated, according to experimental data. Furthermore, we confirmed that YSTB can downregulate the expression of iNOS and ROS, which is consistent with the reduction of NO content. ELISA and PCR results showed that YSTB reversed the LPS-induced levels of pro-inflammatory cytokines TNF-α, IL-6, and IL-1β. In conclusion, we demonstrate that YSTB can successfully inhibit NO activity and reduce the production of pro-inflammatory cytokines TNF-α, IL-6, iNOS, and IL-1β in LPS stimulation within a safe range for YSTB to maintain RAW264.7 cells viability. It demonstrates that YSTB has a definite anti-inflammatory effect on RAW264.7 cells.

With confirming the inhibitory effect of YSTB on CIA mice and RAW264.7 cells, we further analyzed the possible molecular mechanisms involved. According to recent research, the JAK/STAT pathway is closely linked to the development of RA (28). JAK2/STAT3 is an important subtype of JAK/STAT, which can induce systemic inflammatory responses and regulate immunity, closely related to RA disease progression (29). Inflammatory cytokines bind to cell membrane receptors, resulting in the phosphorylation and activation of JAK2 in cells. Then, activated JAK2 triggers phosphorylation of STAT3 protein and further dimerizes STAT3. Dimerized STAT3 protein translocates to the nucleus and induces transcription of target genes involved in synovial cell proliferation, differentiation, and cartilage degradation and expression of numerous pro-inflammatory cytokines, causing a cascade of reactions through autocrine and paracrine signals to form feedback loops that exacerbate disease. At the same time, under the stimulation of lipopolysaccharide, macrophages can significantly activate the JAK2/STAT3 pathway, encoding inflammatory mediators such as TNF-α, IL-1β and IL-6, and different macrophage phenotypes (30). Furthermore, chronic stimulation of these cytokines causes iNOS overexpression, which results in abnormal NO secretion. As a second letter, ROS can also activate the JAK/STAT pathway, causing pro-inflammatory factors to be released and inflammatory mediators to be produced, ultimately leading to pathological damage, toxicity, and apoptosis (31). However, JAK3 is highly expressed in lymphocytes, and the absence or dysfunction of JAK3 will cause lymphocytes to fail to function normally, and eventually lead to the loss of immune function (32). SOCS3 protein is a key negative regulator of this pathway. Once SOCS3 is expressed, it will competitively antagonize JAK2, thereby inhibiting the catalytic activity of JAK2, down-regulating STAT3 phosphorylation expression, and specifically negatively regulating the JAK2/STAT3 pathway, accordingly blocking the enhanced *in vivo* cytokine effect. A previous study found that CIA mice overexpressing SOCS3 had significantly reduced arthritis severity and IL-6 production (33). Furthermore, SOCS3 deficiency increases CD4^+^T cell production of IL-17 and promotes IL-1β induced inflammatory arthropathy.

We looked at the protein expression levels of JAK2, JAK3,STAT3, and SOCS3 in CIA mice and RAW264.7 cells to see if there was a link between the anti-arthritic effect of YSTB and the JAK/STAT3 signaling pathway. In synovial tissue and RAW264.7 cells, YSTB decreased the phosphorylated expression of JAK2, JAK3, and STAT3 proteins while increasing the protein expression level of their downstream protein SOCS3. It is proposed that the ability of YSTB to slow the progression of rheumatoid arthritis may be related to modulating the JAK/STAT pathway by degrading the phosphorylated expression of JAK2, JAK3, and STAT3 proteins, whereas elevated SOCS3 protein expression.

Studies have shown that NF-κB is a classical inflammatory signaling pathway, and P65 is the most representative protein for the regulation and function of NF-κB. MAPK (whose subunits include p38 MAPK, ERK, and JNK, etc.) is an upstream regulator of NF-κB, and with the activation of MAPKs and NF-κB signaling pathways, it accelerates LPS-induced inflammatory responses, which in turn induces the secretion of IL-1β, IL-6 and TNF-α secretion. The results of RAW264.7 cell experiment showed that YSTB significantly reduced the phosphorylation of NF-κB p65, p38 MAPK and ERK protein, but had no significant inhibitory effect on p-JNK.

However, there are still some limitations in the present study. Firstly, although western blotting can reflect proteins expression in RAW264.7 cells, immunofluorescence detection of JAK/STAT3/SOCS3 expression was more intuitive. Besides, the effect of YSTB on nuclear translocation and DNA binding activity of STAT3 was not investigated in this experiment. Therefore, more experiments need to be performed to confirm the mechanism of YSTB in the inhibition of inflammation. Secondly, we focused on JAK/STAT pathway in this study, but the pharmacodynamic mechanism of YSTB is complex, as there are many kinds of ingredients in this compound. Therefore, the research group will carry out *in vivo* gene knockout in mice and further validate more pathways related to inflammation and bone destruction in the subsequent experimental design.

## 5 Conclusion

**Figure 10:**
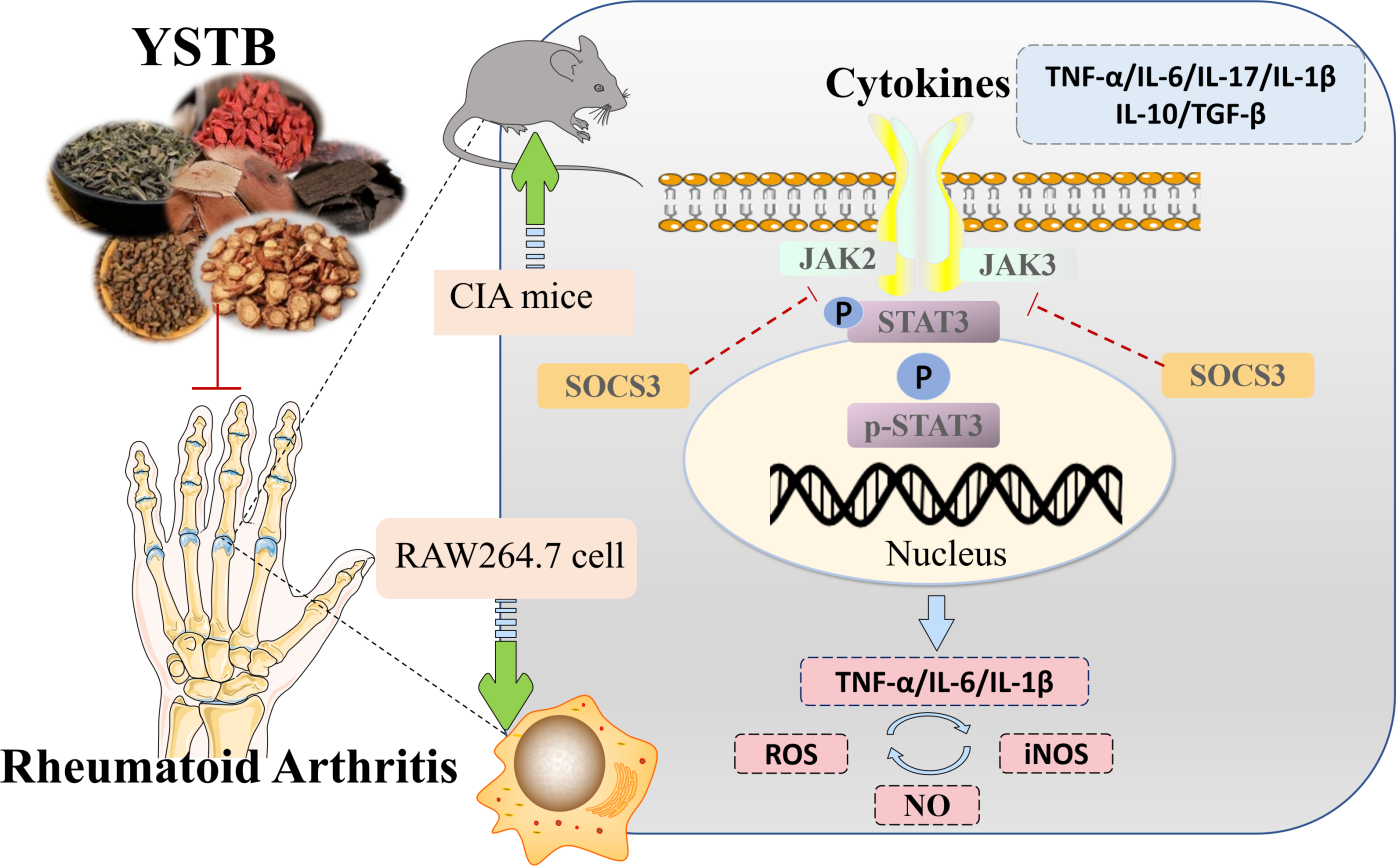
Schematic diagram summarizing the mechanism for YSTB treatment of rheumatoid arthritis.

The present study demonstrated that YSTB had exhibited significant anti-arthritic activity effects on inflammation and bone destruction *in vivo* and *in vitro*, which may be through modulation of the JAK/STAT/SOCS signaling pathway (**Figure 10**) Our current research will contribute to the understanding of YSTB’s probable anti-rheumatoid mechanism and provide a theoretical foundation for its further clinical promotion.

## Data availability statement

The original contributions presented in the study are included in the article/**Supplementary Material**. Further inquiries can be directed to the corresponding author.

## Ethics statement

The animal study was approved by Ethics Committee of Laboratory Animals in The First Affiliated Hospital of Guangzhou University of Chinese Medicine. The study was conducted in accordance with the local legislation and institutional requirements.

## Author contributions

JX: Writing – original draft, Visualization, Validation, Methodology, Investigation, Formal analysis, Data curation, Conceptualization. WJ: Writing – original draft, Software, Methodology, Investigation. D-BW: Writing – original draft, Software, Methodology, Investigation. J-HY: Writing – original draft, Software, Methodology, Investigation. L-JL: Writing – original draft, Validation, Software, Formal analysis, Data curation. M-YZ: Writing – original draft, Validation, Software, Formal analysis, Data curation. G-XC: Writing – review & editing, Supervision, Project administration, Funding acquisition, Conceptualization.
