# Supplementary figures and images for: Yishen Tongbi decoction attenuates inflammation and bone destruction in rheumatoid arthritis by regulating JAK/STAT3/SOCS3 pathway
Source: Front Immunol. 2024 Jun 20;15:1381802. doi: 10.3389/fimmu.2024.1381802 (PMC11222394; doi:10.3389/fimmu.2024.1381802)

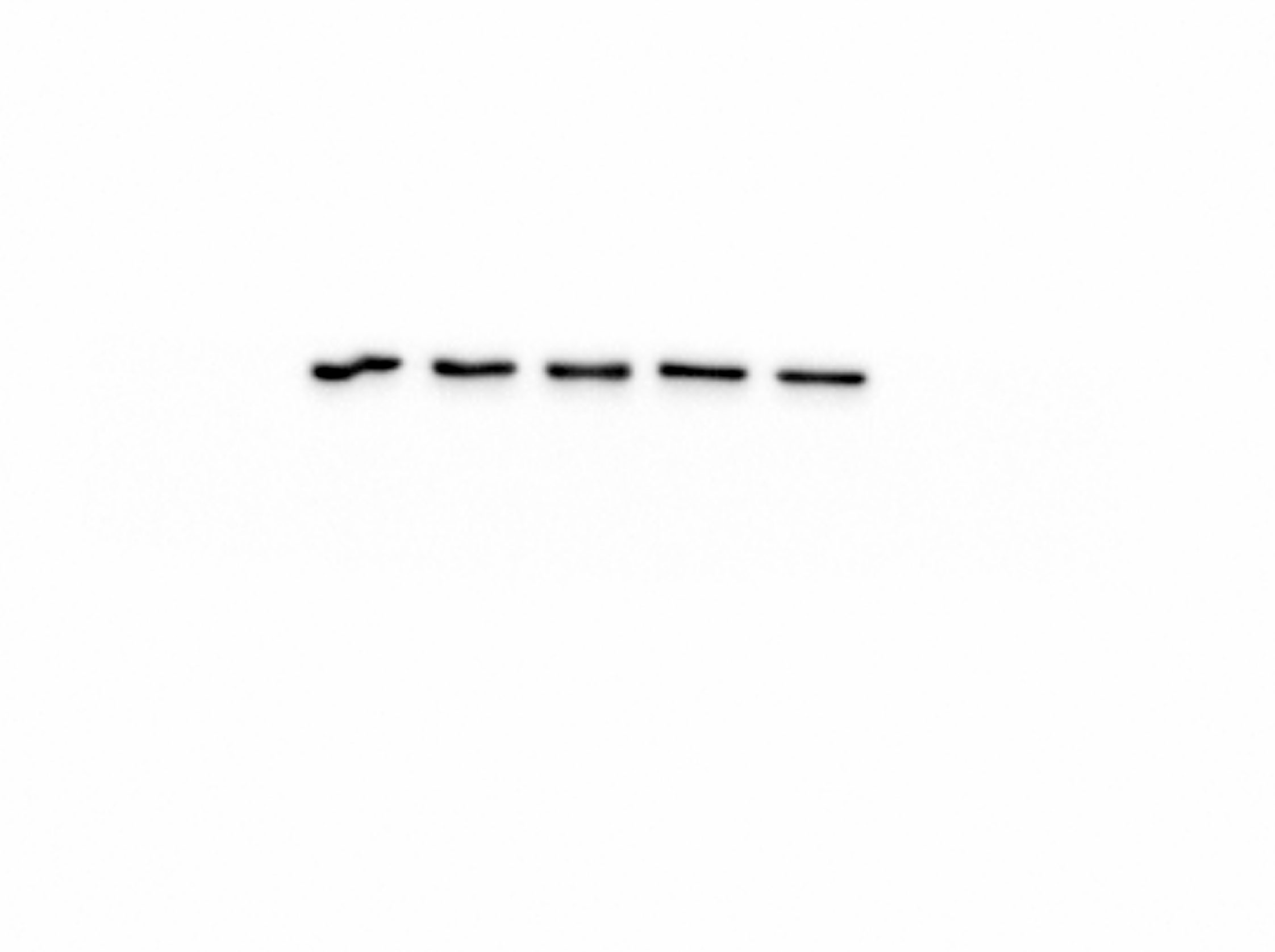

Supplement: Supplementary file 1 [file DataSheet_1.zip › WesternBlot/GAPDH/GAP2.tif]

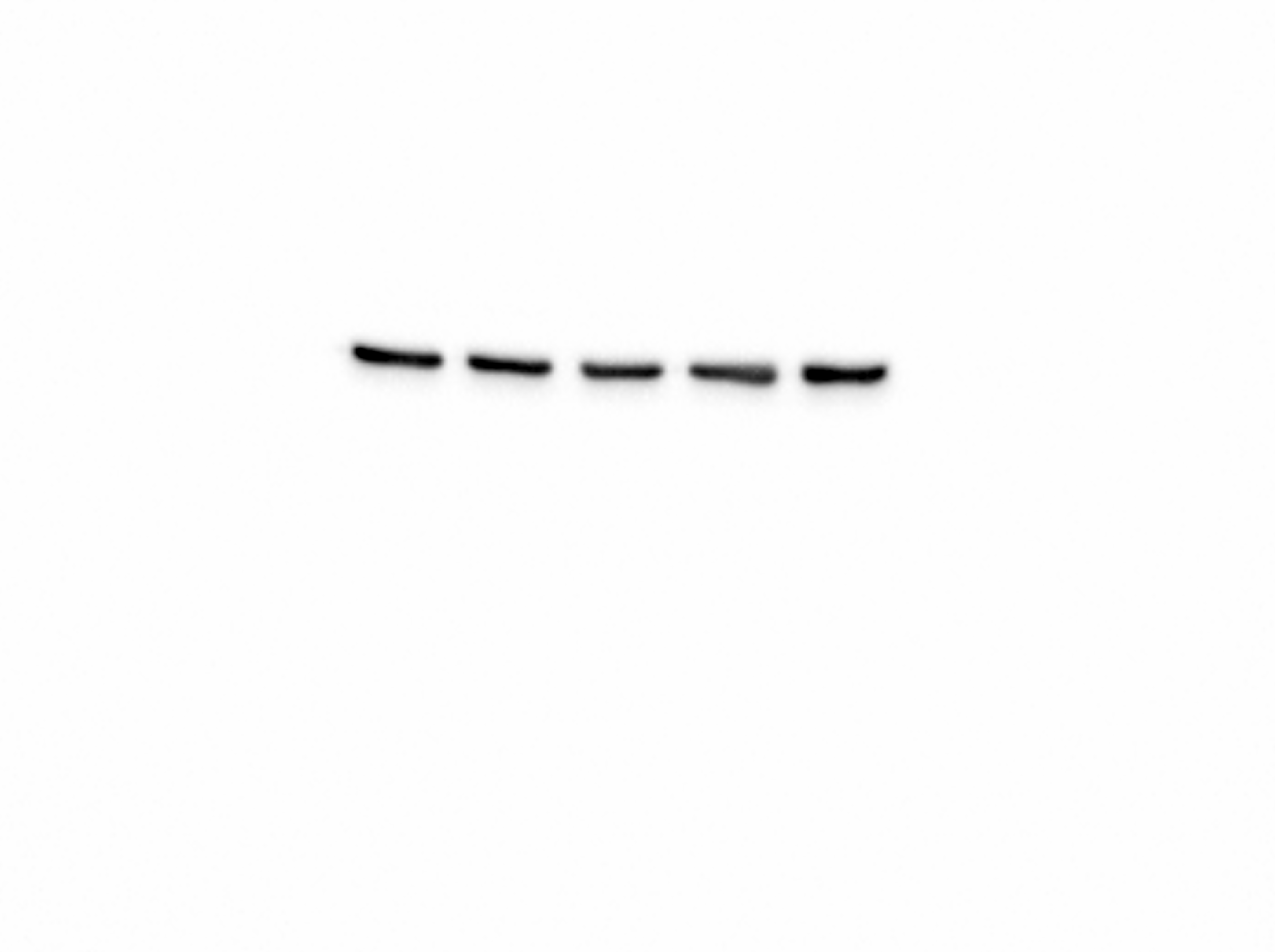

Supplement: Supplementary file 1 [file DataSheet_1.zip › WesternBlot/GAPDH/GAP3.tif]

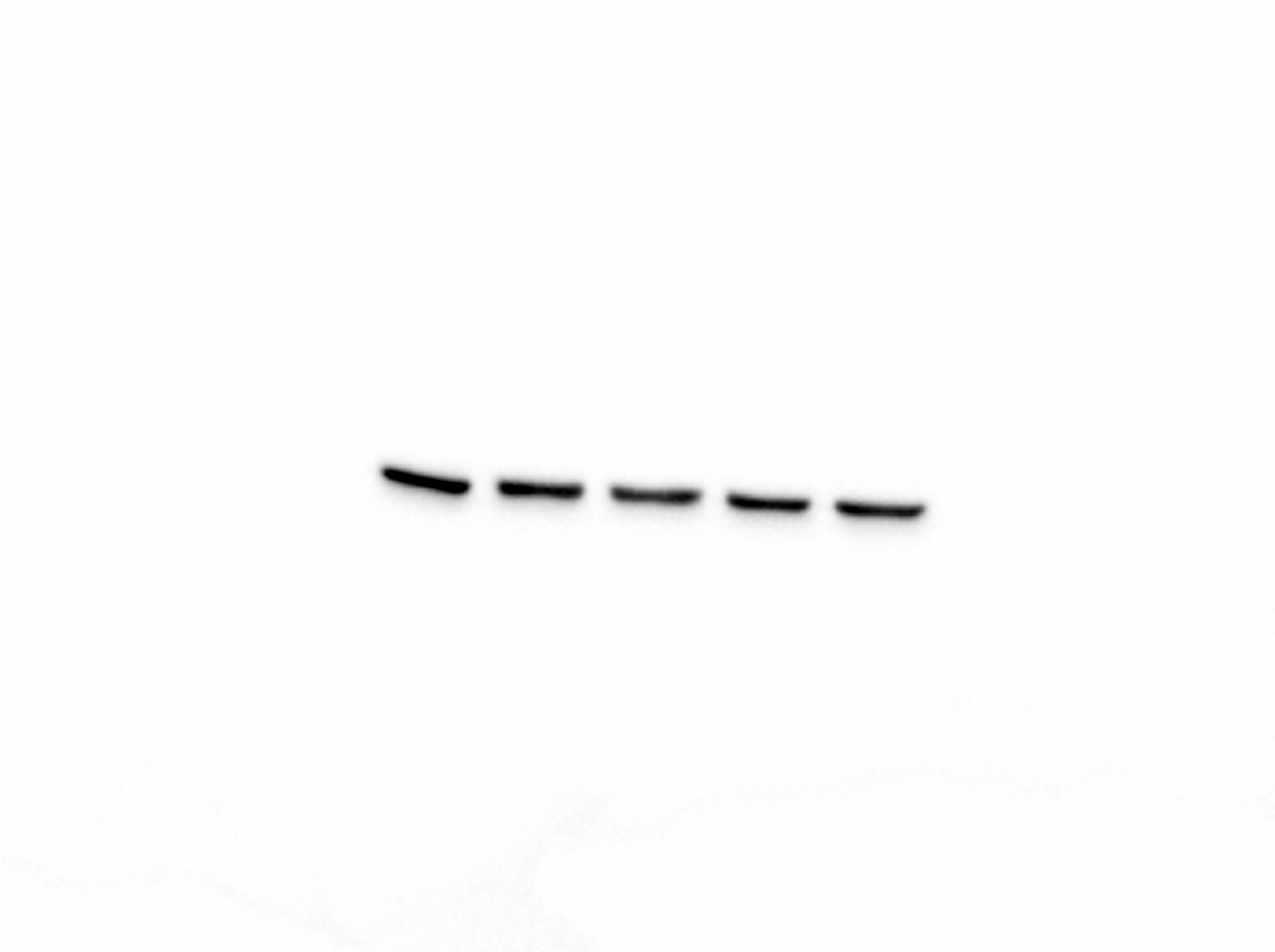

Supplement: Supplementary file 1 [file DataSheet_1.zip › WesternBlot/GAPDH/GAP5.tif]

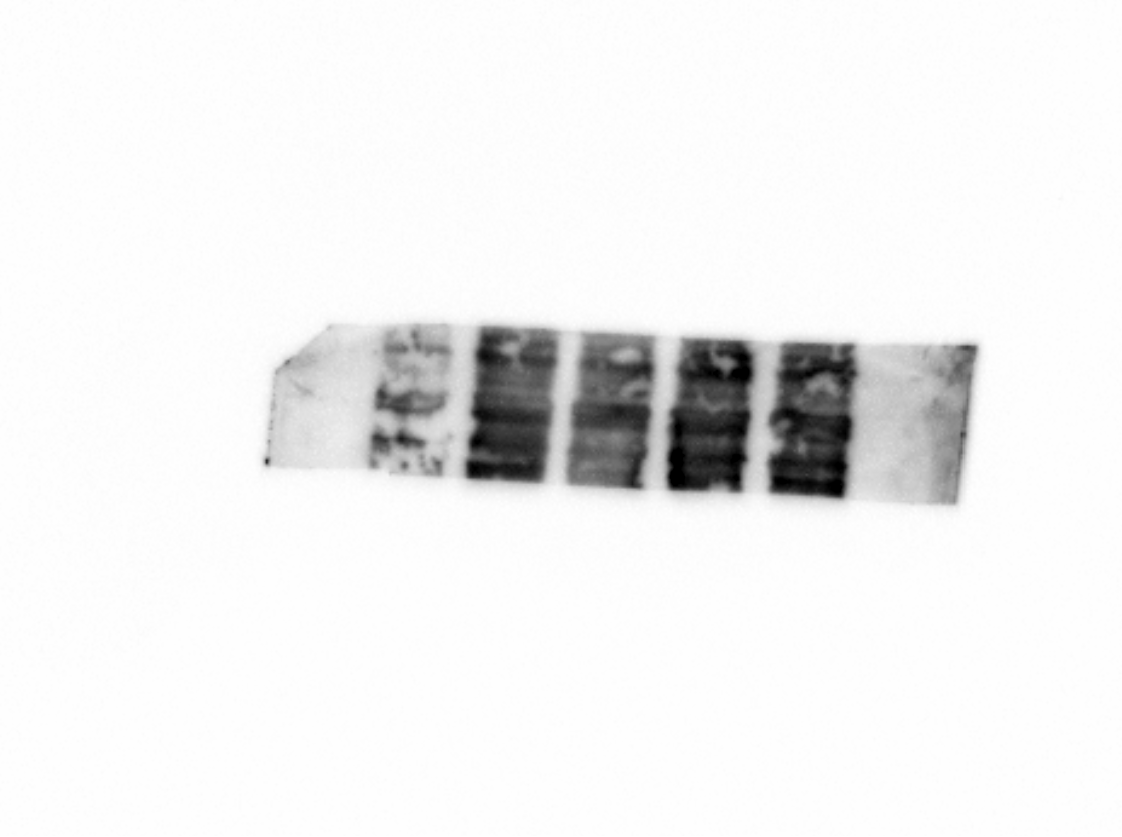

Supplement: Supplementary file 1 [file DataSheet_1.zip › WesternBlot/p-ERK/p-ERK_3.tif]

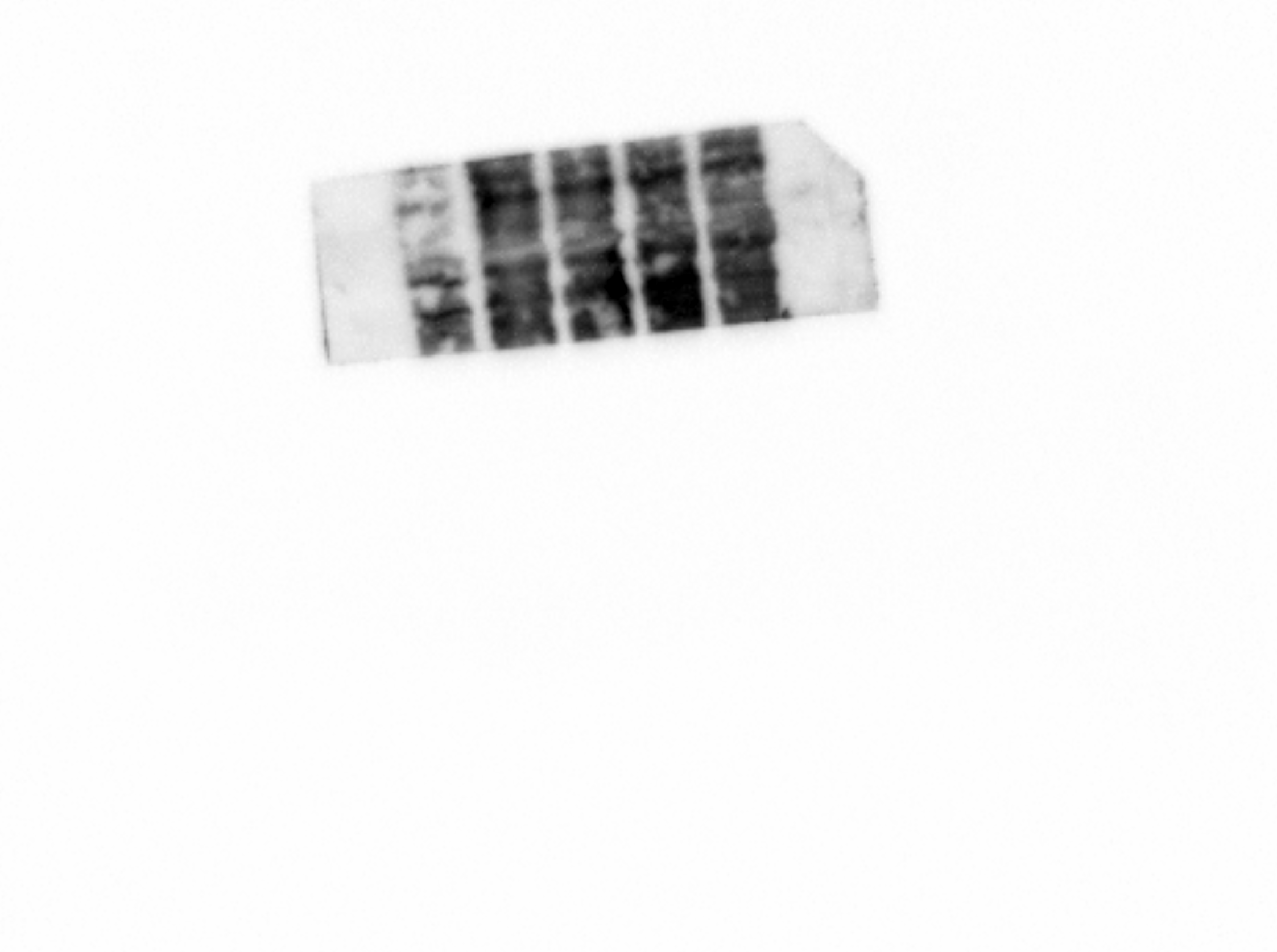

Supplement: Supplementary file 1 [file DataSheet_1.zip › WesternBlot/p-ERK/p_ERK_1.tif]

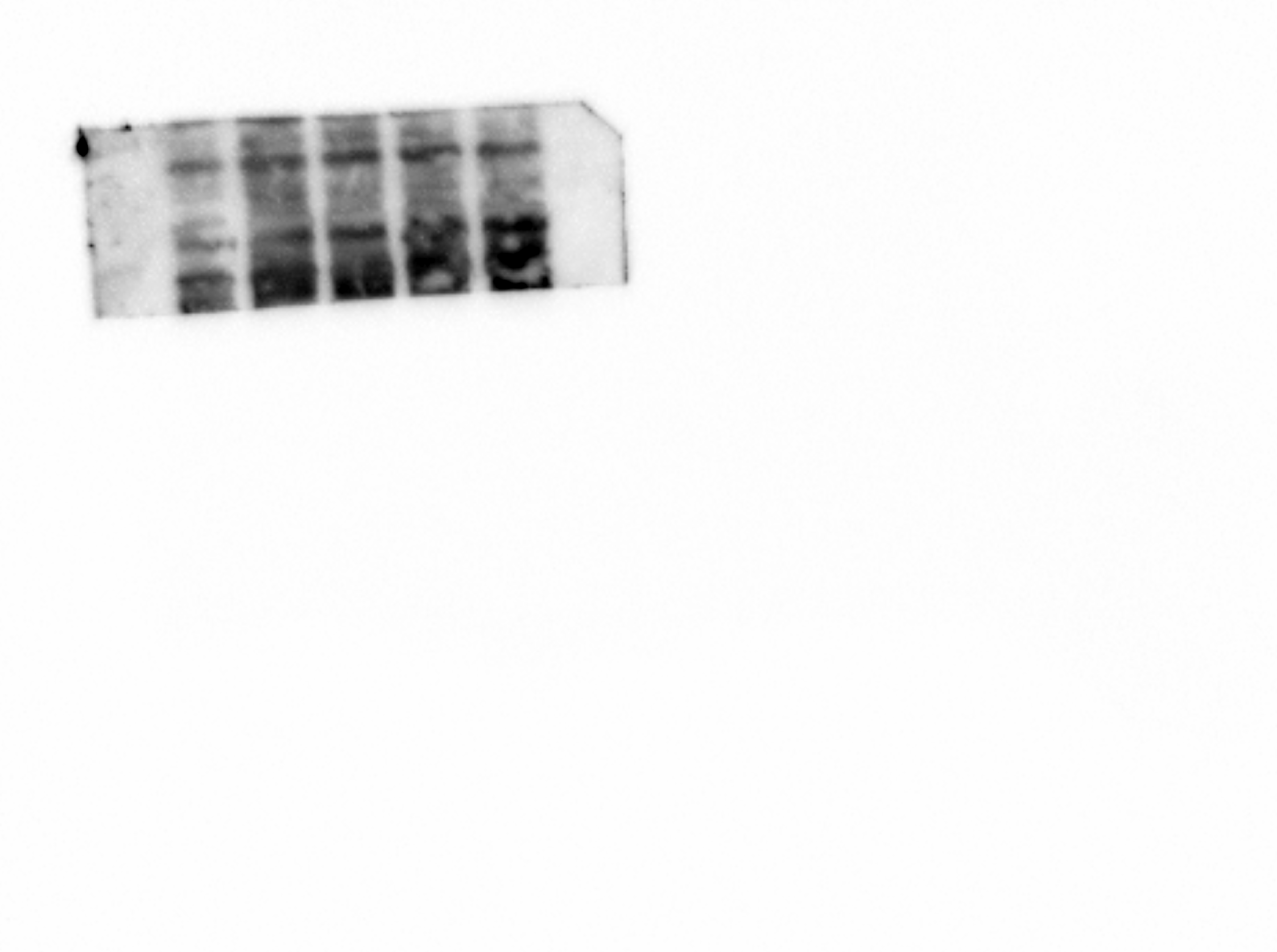

Supplement: Supplementary file 1 [file DataSheet_1.zip › WesternBlot/p-ERK/p_ERK_2.tif]

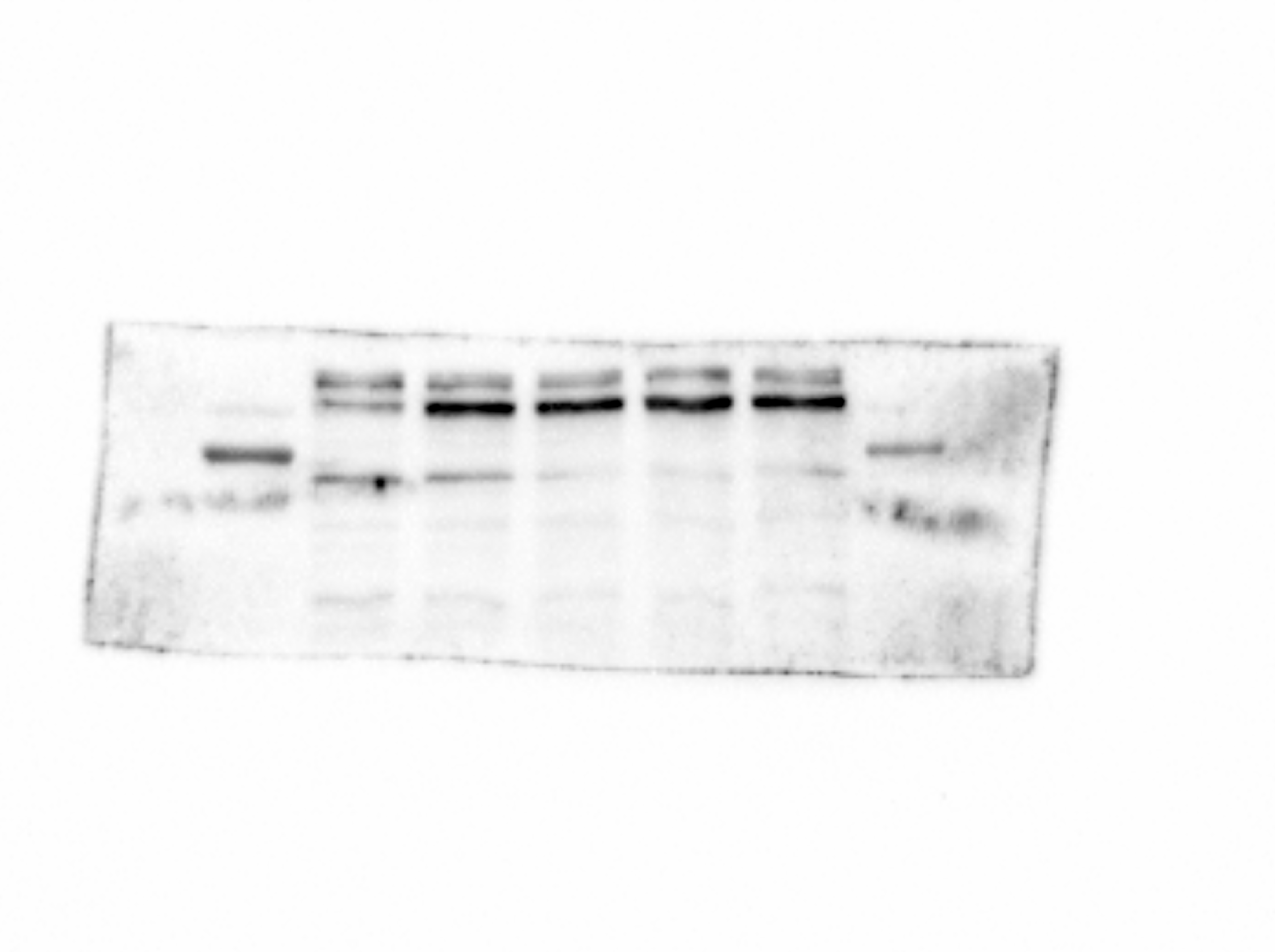

Supplement: Supplementary file 1 [file DataSheet_1.zip › WesternBlot/p-JNK/p-JNK-1.tif]

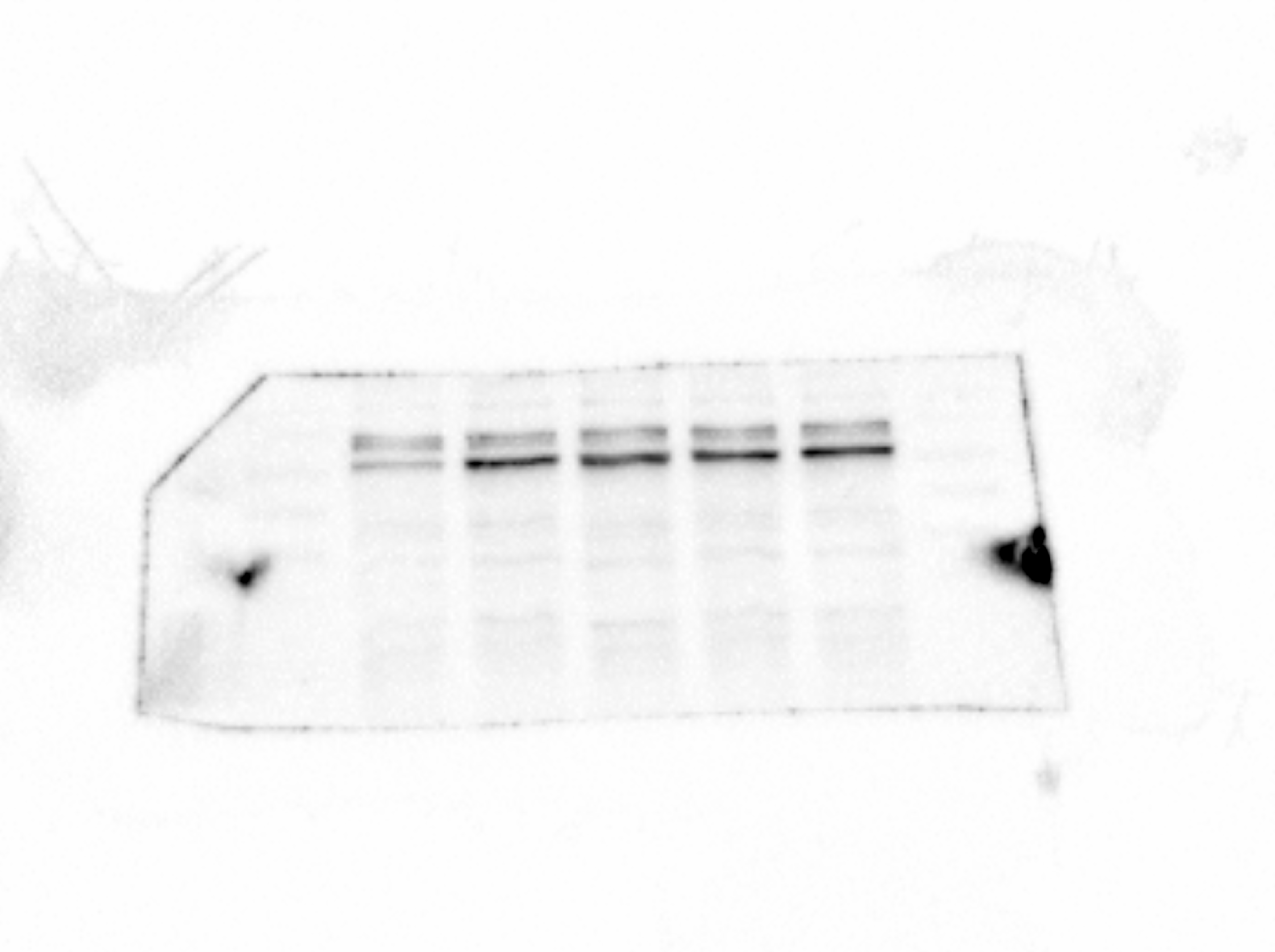

Supplement: Supplementary file 1 [file DataSheet_1.zip › WesternBlot/p-JNK/p-JNK-2.tif]

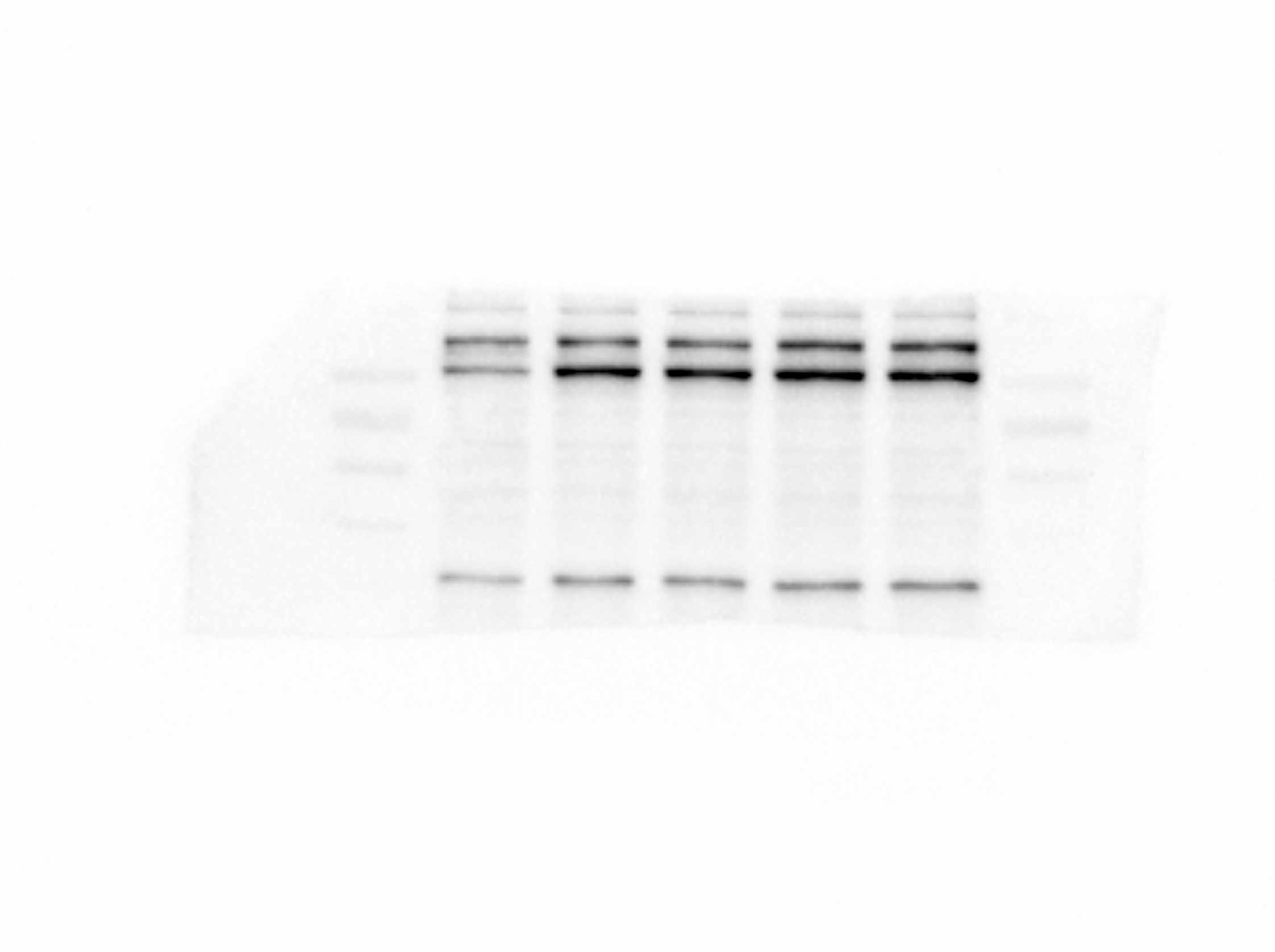

Supplement: Supplementary file 1 [file DataSheet_1.zip › WesternBlot/p-JNK/p-JNK.tif]

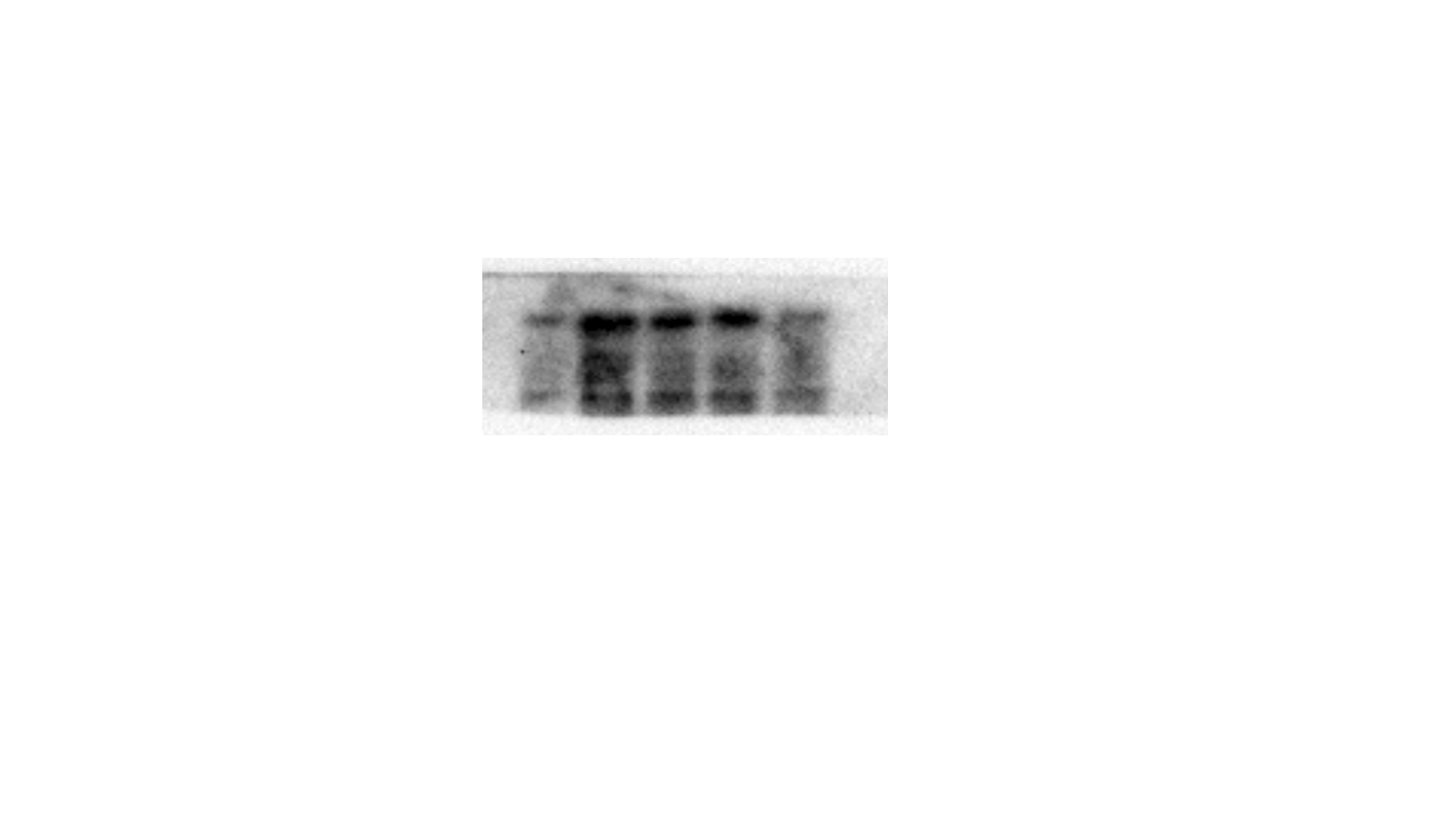

Supplement: Supplementary file 1 [file DataSheet_1.zip › WesternBlot/p-p38/p-p38-1.tif]

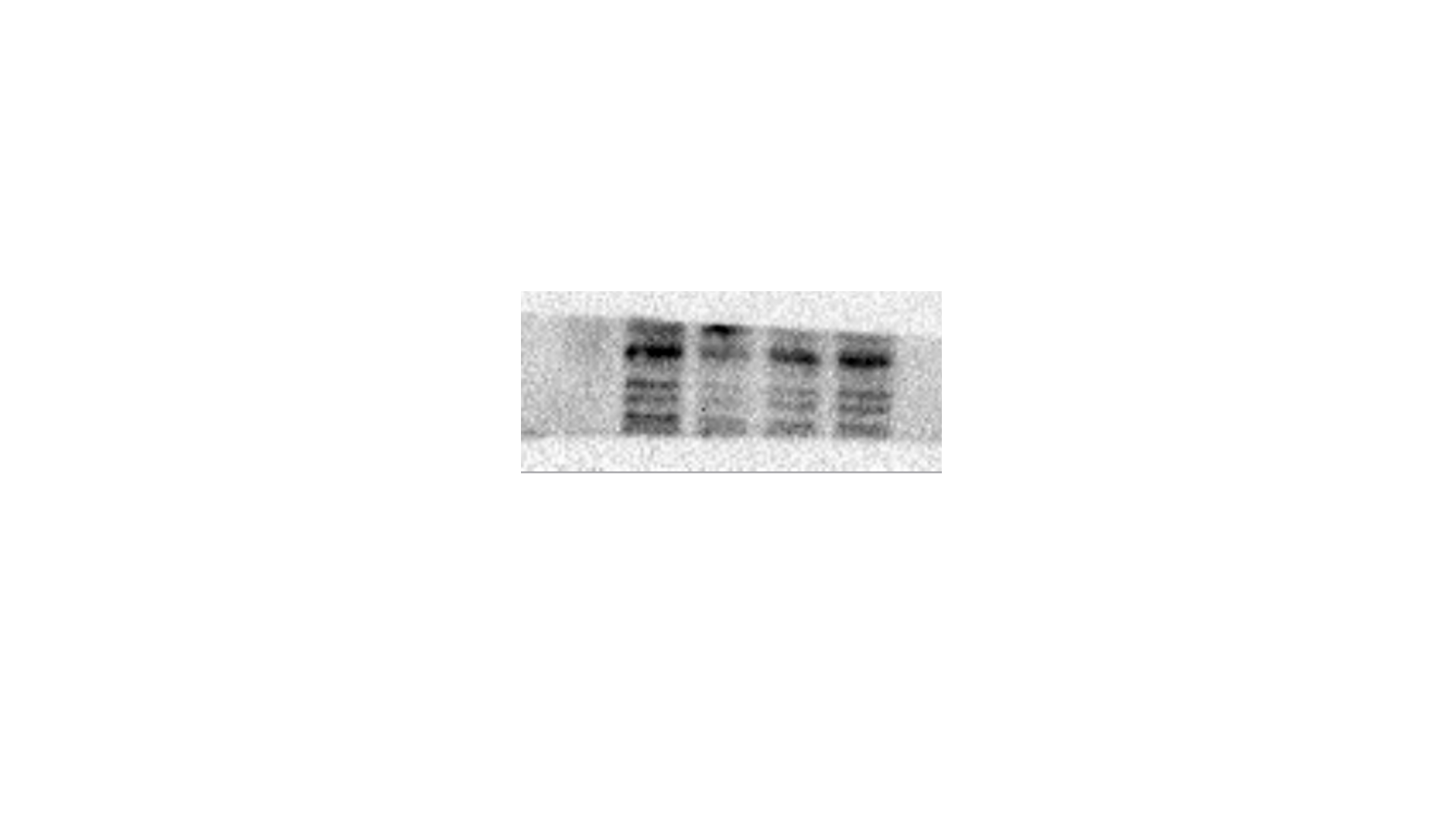

Supplement: Supplementary file 1 [file DataSheet_1.zip › WesternBlot/p-p38/p-p38-2.tif]

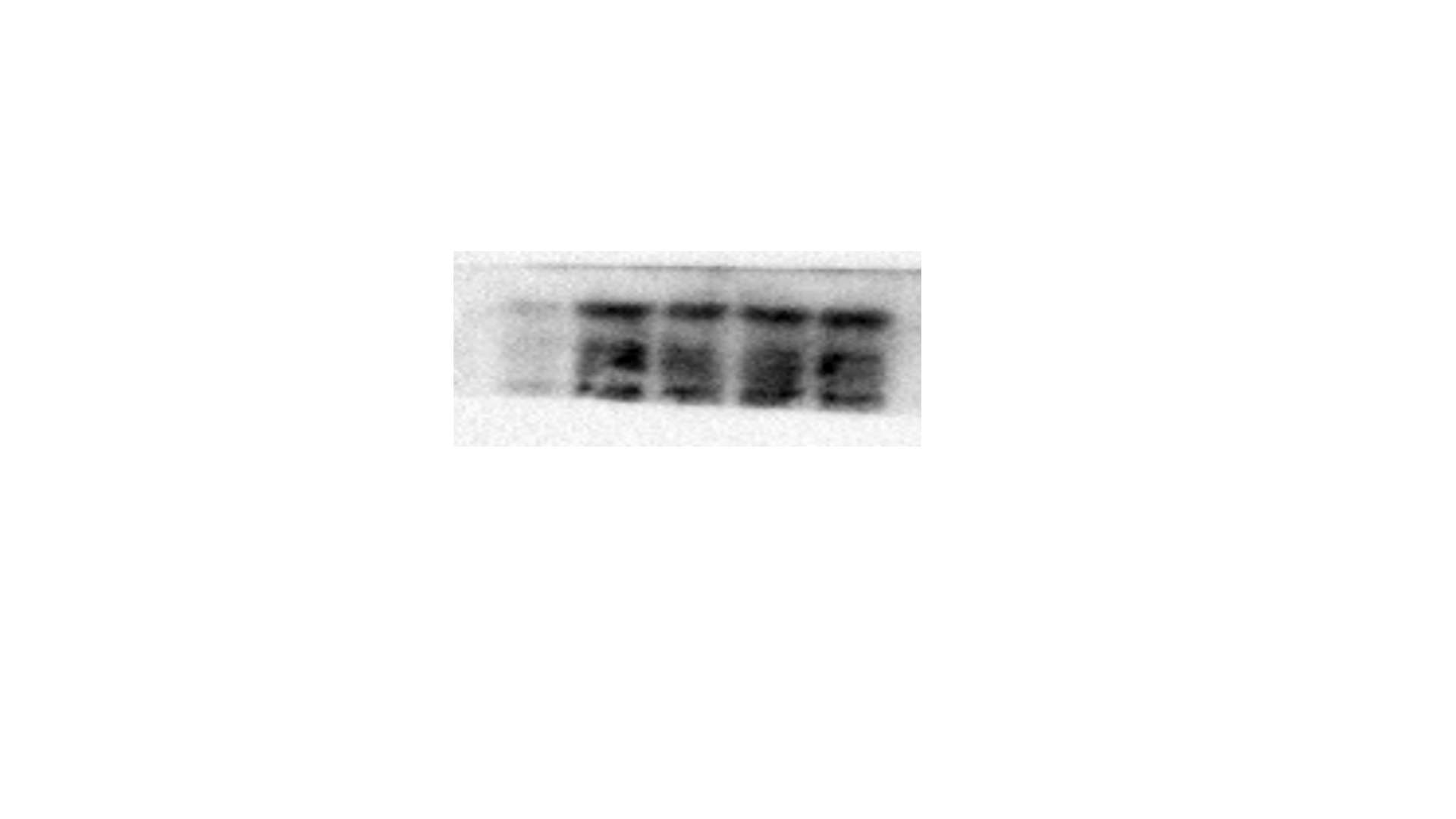

Supplement: Supplementary file 1 [file DataSheet_1.zip › WesternBlot/p-p38/p-p38-3.tif]

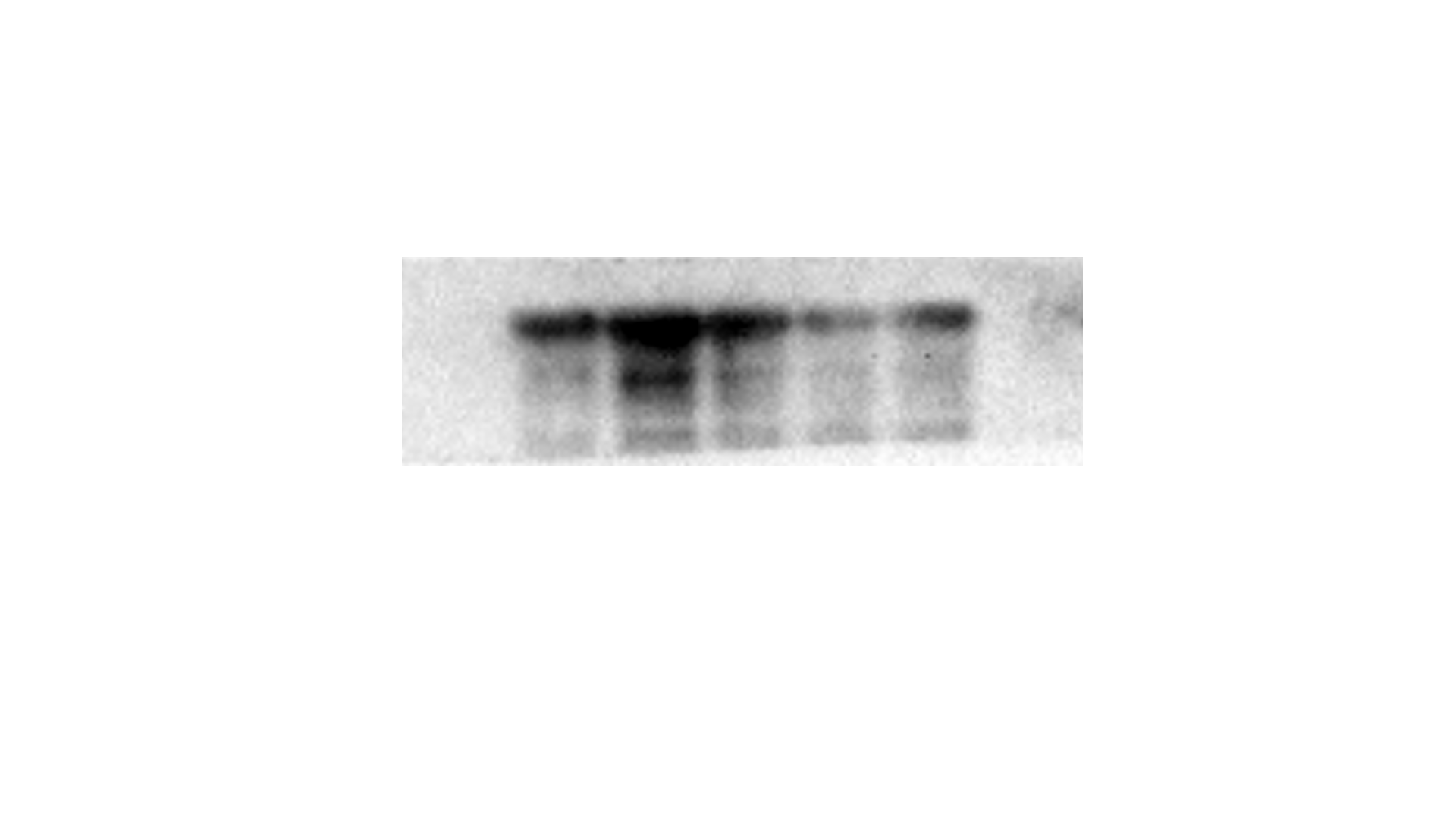

Supplement: Supplementary file 1 [file DataSheet_1.zip › WesternBlot/p-p65/p-p65_1.tif]

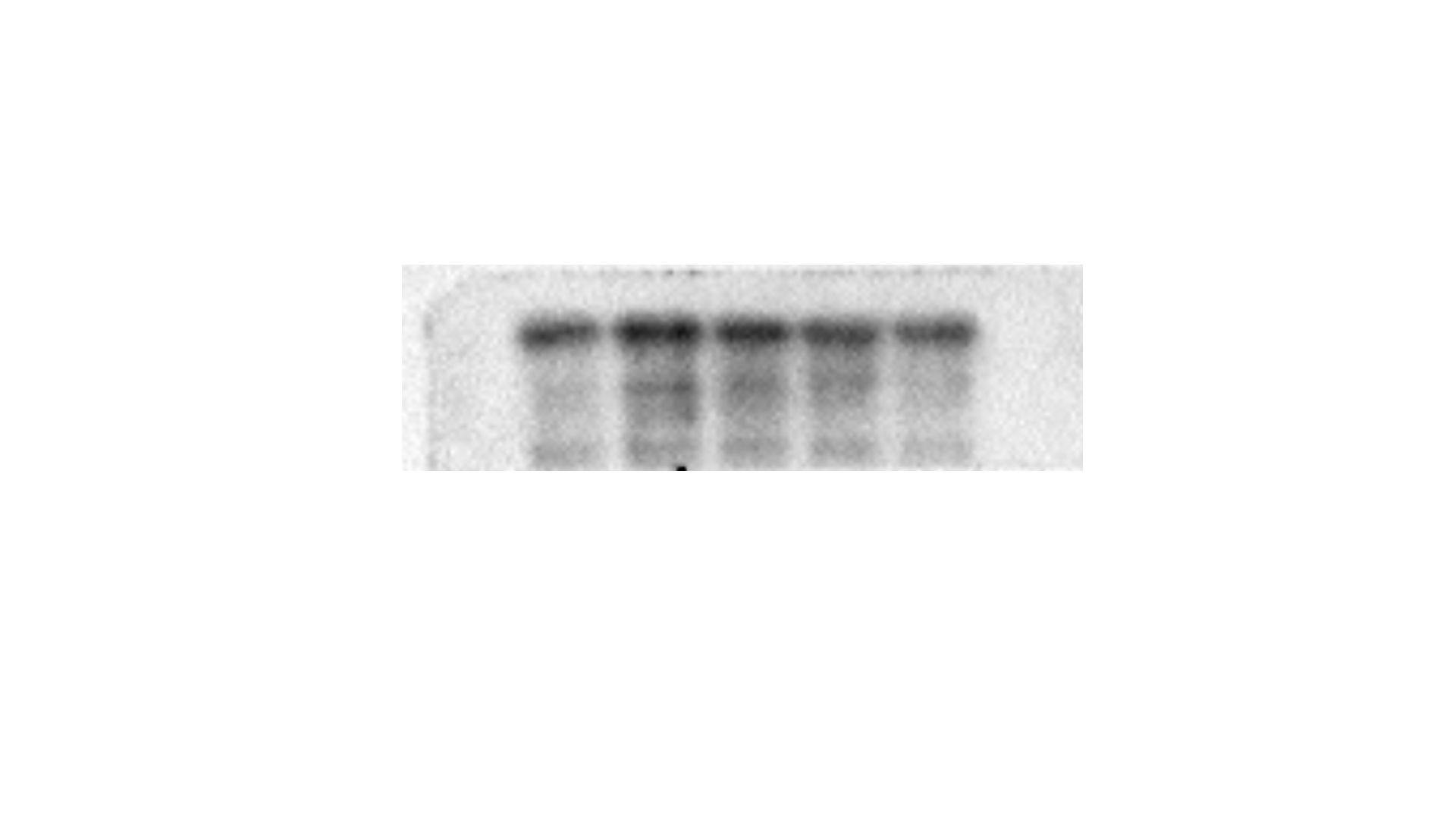

Supplement: Supplementary file 1 [file DataSheet_1.zip › WesternBlot/p-p65/p-p65_2.tif]

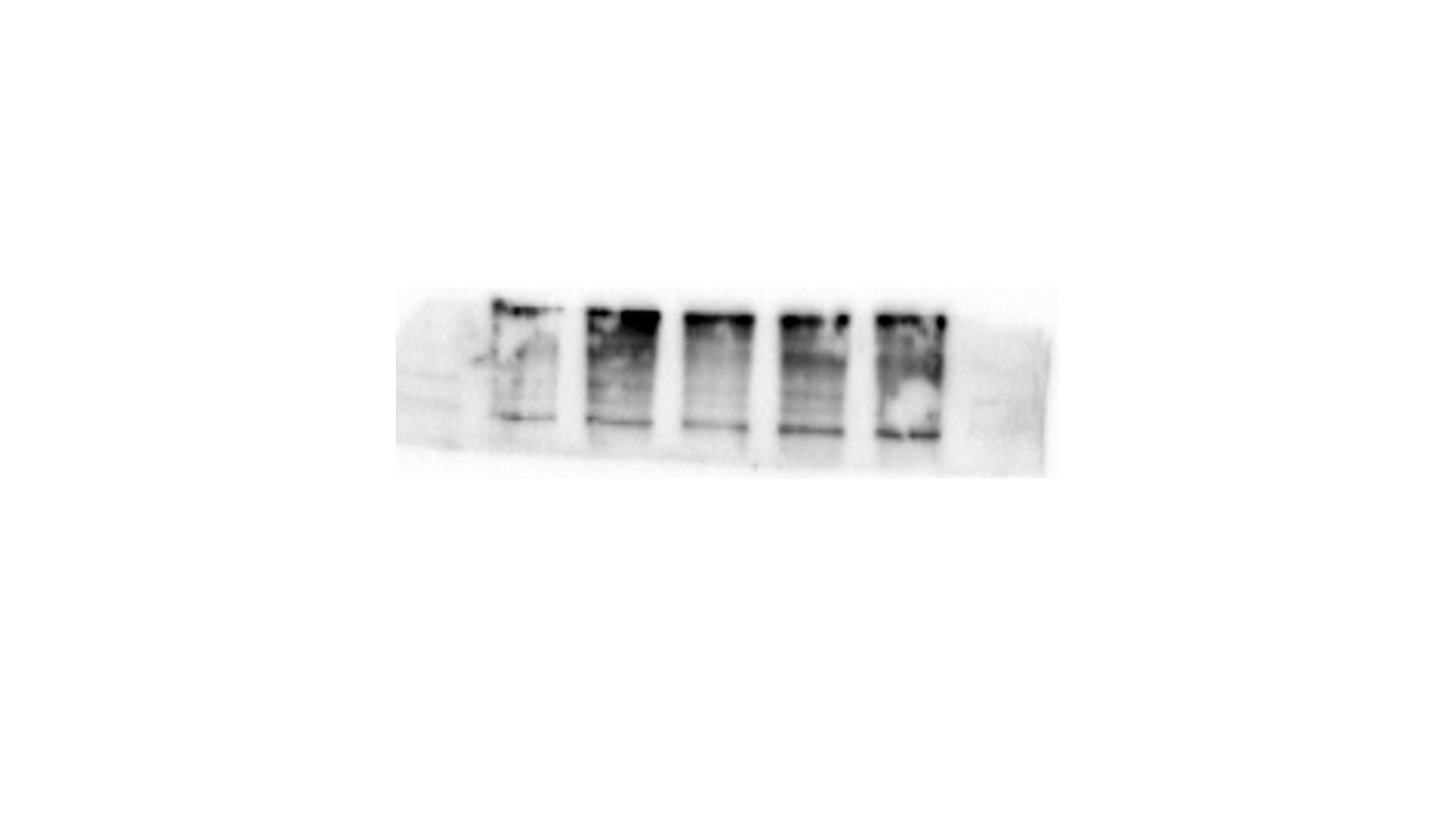

Supplement: Supplementary file 1 [file DataSheet_1.zip › WesternBlot/p-p65/p-p65_3.tif]
